# Single‐Atom Catalyst Aggregates: Size‐Matching is Critical to Electrocatalytic Performance in Sulfur Cathodes

**DOI:** 10.1002/advs.202103773

**Published:** 2021-11-16

**Authors:** Xiaodong Meng, Xing Liu, Xueying Fan, Xin Chen, Shang Chen, Yongqiang Meng, Manyun Wang, Ji Zhou, Song Hong, Lei Zheng, Guosheng Shi, Christopher W. Bielawski, Jianxin Geng

**Affiliations:** ^1^ State Key Laboratory of Organic‐Inorganic Composites Beijing Advanced Innovation Center for Soft Matter Science and Engineering Beijing University of Chemical Technology 15 North Third Ring Road East, Chaoyang District Beijing 100029 China; ^2^ State Key Laboratory Advanced Special Steel Shanghai Applied Radiation Institute Shanghai University Shanghai 200444 China; ^3^ Beijing Synchrotron Radiation Facility Institute of High‐Energy Physics Chinese Academy of Sciences Beijing 100049 China; ^4^ Center for Multidimensional Carbon Materials (CMCM) Institute for Basic Science (IBS) Ulsan 44919 Republic of Korea; ^5^ Department of Chemistry Ulsan National Institute of Science and Technology (UNIST) Ulsan 44919 Republic of Korea; ^6^ Present address: Department of Material Science and Engineering Tiangong University No. 399 BinShuiXi Road, XiQing District Tianjin 300387 China

**Keywords:** aggregates, electrocatalysis, graphene, Li–S batteries, single atom catalysts (SACs)

## Abstract

Electrocatalysis is critical to the performance displayed by sulfur cathodes. However, the constituent electrocatalysts and the sulfur reactants have vastly different molecular sizes, which ultimately restrict electrocatalysis efficiency and hamper device performance. Herein, the authors report that aggregates of cobalt single‐atom catalysts (SACs) attached to graphene via porphyrins can overcome the challenges associated with the catalyst/reactant size mismatch. Atomic‐resolution transmission electron microscopy and X‐ray absorption spectroscopy measurements show that the Co atoms present in the SAC aggregates exist *as single atoms* with spatially resolved dimensions that are commensurate the sulfur species found in sulfur cathodes and thus fully accessible to enable 100% atomic utilization efficiency in electrocatalysis. Density functional theory calculations demonstrate that the Co SAC aggregates can interact with the sulfur species in a synergistic manner that enhances the electrocatalytic effect and promote the performance of sulfur cathodes. For example, Li–S cells prepared from the Co SAC aggregates exhibit outstanding capacity retention (i.e., 505 mA h g^–1^ at 0.5 C after 600 cycles) and excellent rate capability (i.e., 648 mA h g^−1^ at 6 C). An ultrahigh area specific capacity of 12.52 mA h cm^−2^ is achieved at a high sulfur loading of 11.8 mg cm^–2^.

## Graphical abstract

Aggregates of Co single‐atom catalysts (SACs) on graphene are synthesized from a Co(II) porphyrin complex and graphene oxide. The Co atoms in the aggregates exist with spatially‐resolved dimensions that are commensurate with the sulfur species found in sulfur cathodes. As a result, the Co SAC aggregates synergistically interact with the sulfur species and thus maximize electrocatalytic effect in sulfur cathodes.

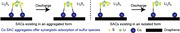

## 1 Introduction

Graphene holds great promise for constructing high‐performance functional materials because of its extraordinary physical and chemical properties, such as a conjugated electronic structure, an ultra large specific surface area, and tunable surface chemistry.^[^ ^1^ ^]^ Single‐atom catalysts (SACs) immobilized on graphene, particularly those that employ Ni, Co, or Fe, show excellent electrocatalytic activities in the hydrogen evolution reaction (HER),^[^ ^2^ ^]^ the CO_2_ reduction reaction,^[^ ^3^ ^]^ and the oxygen evolution reaction (OER).^[^ ^4^ ^]^ The synthesis of graphene‐based SACs typically consists of three steps: mixing a suspension of graphene oxide (GO) with an aqueous solution of an inorganic metal salt, freeze‐drying the combined suspension, and then thermally annealing the obtained composite under the reducing atmosphere of ammonia. In the last step, ammonia provides a means to simultaneously dope the graphene with beneficial heteroatoms (i.e., nitrogen) while complexing the single‐atom metals.^[^ ^2^, ^3^, ^4^, ^5^ ^]^ Such processes are often challenged by the use of a toxic gas as the heteroatom source and the tendency of the metal atoms to form clusters or even larger particles during the annealing step.^[^ ^6^ ^]^ The difficulty can be expected to be managed through molecular design as the unique surface of graphene provides opportunities to form *π*‐stacking interactions with conjugated molecules such as pyrene, perylene bisimide, polythiophene, and, notably, porphyrins.^[^ ^7^ ^]^ Such noncovalent approaches are attractive when compared to their covalent counterparts as the former do not affect the *π*‐conjugated structures of graphene and can even mitigate the structural defects that are often found on the basal planes of graphene.^[^ ^8^ ^]^ Indeed, graphene‐based composites that are prepared via noncovalent interactions have been employed in a number of applications, including sensing,^[^ ^7^, ^9^ ^]^ photothermal systems,^[^ ^7e^ ^]^ lithium‐ion batteries,^[^ ^7b^ ^]^ OER chemistry,^[^ ^10^ ^]^ and the oxygen reduction reaction.^[^ ^10^, ^11^ ^]^

Lithium–sulfur (Li–S) batteries are often regarded as next‐generation energy storage devices because of their high energy density (2600 W h kg^–1^) as well as the high abundance and environmental friendliness of sulfur.^[^ ^12^ ^]^ However, their commercial applications are severely restricted by two critical factors: 1) a loss in the active material sulfur during iterative charge/discharge cycles due to the “shuttle effect” of the polysulfides and 2) sluggish cathode kinetics.^[^ ^13^ ^]^ Considerable efforts have been employed to overcome these limitations, typically by utilizing electrocatalysts to promote the chemical reactions in sulfur cathodes. So far, a variety of nanostructured materials, including nanoparticles (Co, Pt, Ni, CoS_2_, CoN, CeO_2_, and MgO),^[^ ^13^, ^14^ ^]^ nanotubes (MoS_2_),^[^ ^15^ ^]^ nanorods (TiN, FeOOH),^[^ ^16^ ^]^ nanosheets (MoS_2_, Ti_3_C_2_T, WS_2_),^[^ ^17^ ^]^ nanocubes (Ni_3_N_0.85_),^[^ ^18^ ^]^ nanoribbons (VN),^[^ ^19^ ^]^ and nanoclusters (Nb_2_O_5–_ *_x_*)^[^ ^20^ ^]^ have been incorporated into carbon materials and used as electrocatalysts in sulfur hosts. Since only the exposed surfaces and edges of these electrocatalysts are active, the sulfur host materials typically require a relatively high catalyst loading (often >20 wt%) or even pure catalyst matrices to achieve satisfactory effects.^[^ ^21^ ^]^ By contrast, SACs can be expected to exhibit a 100% atomic utilization efficiency and a number of SACs have been demonstrated to promote the cathode reactions in Li–S batteries.^[^ ^22^ ^]^ However, current SACs typically do not deliver optimal catalytic activities in sulfur cathodes because of a range of intrinsic challenges, including a propensity of the SACs to coalesce during preparation and the size mismatch between the SACs and the relatively large reactants, such as S_8_ and polysulfides, which restricts interaction between the two components. Matching the distances between the metal atoms in the SACs with the molecular sizes of the sulfur species found in sulfur cathodes, while suppressing the detrimental coalescence of the single‐atom metals, can be expected to enhance the electrocatalytic effects and afford Li–S batteries that exhibit superlative performance metrics.

Herein, we report aggregates of Co SACs that feature commensurate size with the sulfur reactants found in sulfur cathodes and show that these catalysts significantly enhance the electrocatalytic effects required by sulfur cathode chemistry. The key material, designated as Co‐NG(800), was obtained by combining a Co(II) porphyrin complex with GO followed by hydrothermal treatment and thermal annealing. A series of high‐angle annular dark field‐scanning transmission electron microscopy (HAADF‐STEM) and X‐ray absorption spectroscopy (XAS) measurements in conjunction with density functional theory (DFT) calculations revealed that the intrinsic coordination structure of the Co(II) porphyrin complex (i.e., Co–N_4_) was preserved and effectively prevented the formation of Co—Co bonds in the aggregates. As a result, the aggregates of the Co SACs promoted the catalytic activity of the Co atoms by enabling synergistic interactions with polysulfides. When used as an electrocatalytic sulfur host, the Co‐NG(800) significantly enhanced the performance of Li–S batteries in terms of specific capacity and rate capacity (1346 and 648 mA h g^−1^ at 0.1 C and 6 C, respectively) as well as a cycling stability (505 mA h g^−1^ after 600 cycles at 0.5 C). Ultrahigh area specific capacities were also measured from cells with high sulfur loadings (e.g., 12.52 mA h cm^−2^ at an area loading of 11.8 mg cm^−2^). In addition to establishing a new approach to solving the longstanding challenges associated with Li–S batteries and to realizing high‐performance devices, the strategy described offers a general guide for adapting SACs for use in a broad range of contemporary applications.

## 2 Results and Discussion

### 2.1 Design and Synthesis of the Co SACs on Graphene

**Figure 1:**
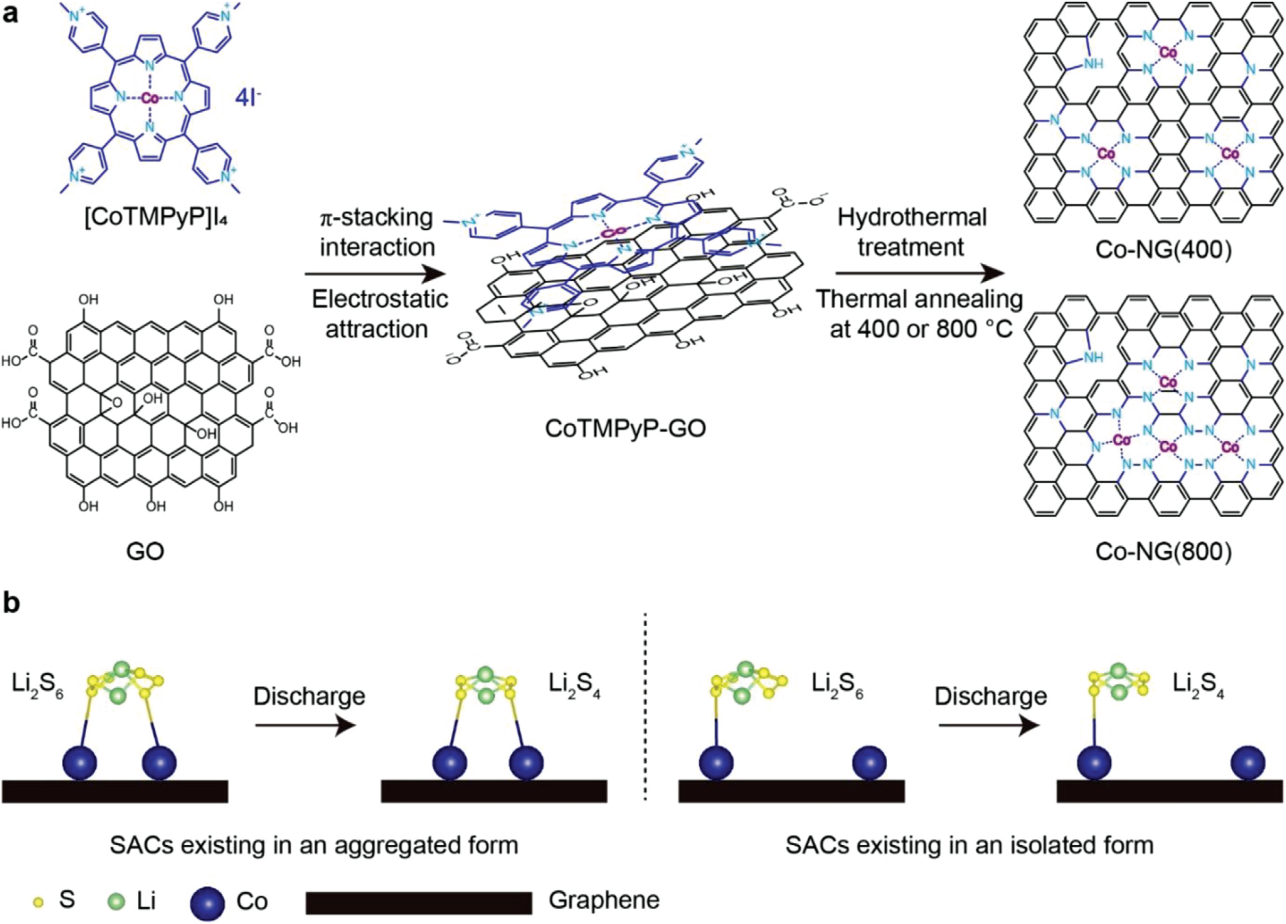
Synthesis of SACs in different forms and an illustration of polysulfide absorption. a) Synthetic route used to prepare Co SACs (i.e., Co‐NG(400) and Co‐NG(800)) using a water‐soluble Co(II) porphyrin complex. b) Illustrations showing different absorption manners of polysulfides to SACs that exist in either aggregated (left) or isolated (right) forms.

As summarized in **Figure** **1**a, Co SACs were synthesized from cobalt(II) 5,10,15,20‐meso‐tetrakis(*N*‐methyl‐4‐pyridinyl)porphyrin tetraiodide ([CoTMPyP]I_4_), a water‐soluble Co(II) porphyrin complex. First, an aqueous solution of [CoTMPyP]I_4_ was mixed with an aqueous suspension of GO. A series of spectroscopic and Zeta potential measurements revealed that the porphyrins were attached to the GO sheets via *π*‐stacking interaction and electrostatic attraction (Figures S2 and S3, Supporting Information), which were expected since the porphyrin features a *π*‐conjugated structure and positively charged amino groups that, collectively, are complementary to the functional groups present on GO. The resulting composite (designated as CoTMPyP‐GO) was hydrothermally treated at 180 °C and then thermally annealed at 400 or 800 °C to convert GO into graphene, which was simultaneously doped with heteroatom N and loaded with Co atoms during the processes. The products were designated as Co‐NG(400) and Co‐NG(800), depending on the annealing temperature. As will be shown below, the use of the higher annealing temperature facilitated transformation of the Co SACs to an aggregated form that differed from those found in metal clusters or nanoparticles (Figure 1a). The result can be ascribed to the porphyrin ligand which effectively coordinates the metal in a multidentate fashion and, as such, bestows stability, particularly at elevated temperatures. Controls included N‐doped graphene (designated as NG), which was prepared in a similar fashion as Co‐NG(800) except that a porphyrin devoid of Co (i.e., [TMPyP]I_4_) was used, and a graphene (designated as G) that was obtained by repeating the synthesis in the absence of a porphyrin additive.

Macrocyclic S_8_ and various polysulfides (Li_2_S*_x_*, 4 ≤ *x* ≤ 8) are common reactants found in the cathodes of Li–S batteries. These species are larger than those found in the HER, OER, O_2_, and CO_2_ reduction reactions (e.g., H_2_, O_2_, and CO_2_).^[^ ^2^, ^3^, ^4^ ^]^ Size matching is a key criterion in SACs as size differentials between the catalysts and the reactants can hamper efficiency, even if the isolated metal atoms are fully exposed. A potential solution to this issue is shown in Figure 1b. Positioning multiple Co atoms over length scales that are commensurate with reactant size may facilitate synergistic interactions between the SACs and the reactants, and thus promote electrocatalysis. As will be shown below, attaching the SACs to graphene in an aggregated form was found to have a profound effect on electrocatalyst performance and viability in sulfur cathodes.

### 2.2 Characterization of the SACs on Graphene

**Figure 2:**
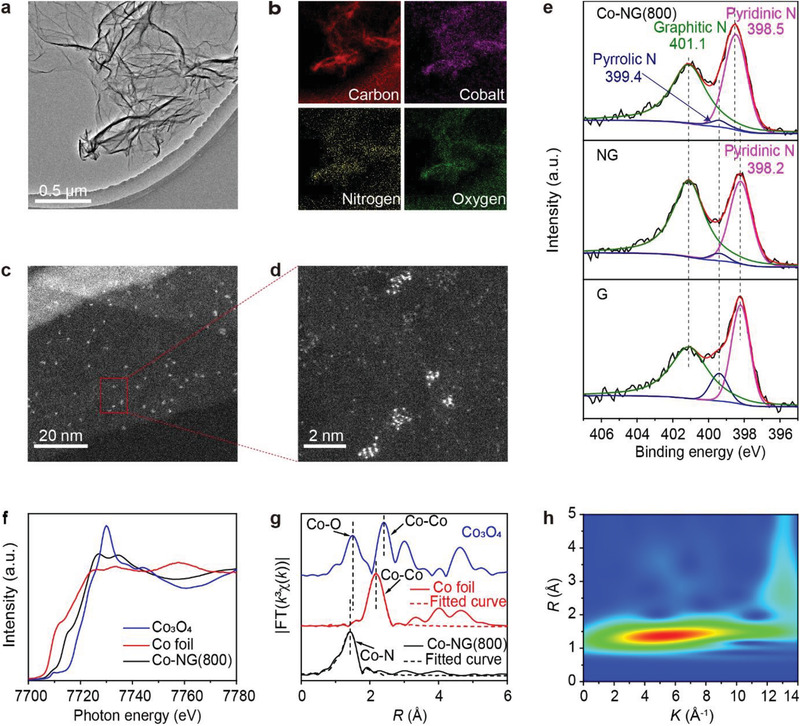
Characterization of the Co‐NG(800) electrocatalyst. a) TEM image of Co‐NG(800) and b) elemental maps collected from the entire area shown in panel (a). c,d) HAADF‐STEM images of the Co‐NG(800) recorded at different magnifications. e) N 1s XPS spectra recorded for Co‐NG(800), NG, and G (indicated). f) Co *K*‐edge XANES spectra recorded for the Co‐NG(800), Co_3_O_4_, and Co foil (indicated). g) FT spectra obtained from the *k* ^3^‐weighted EXAFS data and the corresponding fitted curves (dashed lines, indicated). h) WT contour plots of the EXAFS for Co‐NG(800).

**Figure** **2**a presents a transmission electron microscopy (TEM) image of Co‐NG(800), where the sheet morphology and surface wrinkles can be observed along with the absence of errant particles. The observation was further supported by an X‐ray diffraction analysis which indicated that Co‐containing compounds were lacking (Figure S4, Supporting Information). However, an elemental analysis revealed that cobalt was present and distributions of all the constituent elements were consistent with the morphology of the material (Figure 2b). To clarify, the Co‐NG(800) was subsequently analyzed by aberration‐corrected STEM. An HAADF‐STEM image revealed very bright spots that were uniformly distributed on the Co‐NG(800) sheets (Figure 2c). Given the elemental composition of the Co‐NG(800), these bright spots were assigned to the Co atoms. Increasing the magnification indicated that each bright spot was consisted of multiple Co atoms and the Co SACs effectively formed aggregates (Figure 2d). A statistical analysis of ≈200 aggregates revealed that most of them contained between 5 and 14 Co atoms with aggregates containing 10 Co atoms accounting for the largest fraction (Figure S5, Supporting Information). The distances between two adjacent Co atoms in the aggregates were measured to range between 0.2 and 0.6 nm and those in the largest fraction ranged from 0.25 to 0.45 nm (Figure S6, Supporting Information). The Co SACs may adopt a separate layer on the graphene surfaces by forming intermolecular interactions between the Co‐containing moieties and the underlying graphene.^[^ ^23^ ^]^ Alternatively, the Co SACs may be incorporated into the graphene plane via chemical reactions between the Co porphyrin complex and the GO starting material as GO is known to feature myriad structural defects and functional groups.^[^ ^24^ ^]^

The Co‐NG(800), NG, and G were further investigated using X‐ray photoelectron spectroscopy (XPS). The Co content of Co‐NG(800) was determined to be 2.50 wt%. The N content values measured for Co‐NG(800) and NG were relatively high (5.43 and 4.46 wt%, respectively), particularly when compared to G (2.23 wt%), due to the porphyrin additive used during the synthesis. Deconvolution of the XPS data indicated that pyridinic‐, pyrrolic‐, and graphitic‐type N atoms were present, and that the pyridinic N atoms were dominate. Moreover, the pyridinic N atoms appeared to be complexed to Co as the corresponding binding energy recorded for Co‐NG(800) was found to be 0.3 eV higher than that recorded for NG or G.

The chemical environment of the Co atoms in Co‐NG(800) was further investigated using XAS. As shown in Figure 2f, the Co *K*‐edge X‐ray absorption near‐edge structure (XANES) spectrum recorded for Co‐NG(800) contained a weak pre‐edge feature at 7709 eV, which was assigned to a 1s → 3d transition, as well as a shoulder peak and well‐resolved double absorption‐edge peaks at 7715.2, 7726.9, and 7734.5 eV, which corresponded to 1s → 4p transitions.^[^ ^25^ ^]^ The weak pre‐edge absorption indicated that the Co atoms adopted a centrosymmetric coordination structure.^[^ ^26^ ^]^ In addition, the absorption edge recorded for Co‐NG(800) was located in‐between those recorded for a Co foil (Co^0^) and Co_3_O_4_ (Co^2+^, Co^3+^), and thus enabled the oxidation state of the complexed Co atoms in Co‐NG(800) to be ascertained.^[^ ^22a^ ^]^

Figure 2g shows a series of Fourier‐transformed (FT) spectra in *R* space, as obtained from the *k* ^3^‐weighted extended X‐ray absorption fine structure (EXAFS) data, for Co‐NG(800), Co_3_O_4_, and a Co foil. In contrast to the peaks observed at 1.50 and 2.42 Å, which stemmed from the Co—O and Co—Co bonds in Co_3_O_4_, and the peak at 2.18 Å, which was assigned to the Co—Co bonds in the Co foil, the dominate peak at 1.41 Å in the spectrum recorded for the Co‐NG(800) was attributed to the presence of Co—N bonds. Fitting the EXAFS spectra enabled a coordination number of four (i.e., Co—N_4_) to be assigned to the Co atoms in Co‐NG(800) (Table S1, Supporting Information). Furthermore, a wavelet transform (WT) analysis of the EXAFS spectra was performed to increase resolution in the *k* and *R* spaces. In contrast to the two intensity maxima at 7.0 and 6.6 Å^–1^ stemming from Co—O and Co—Co contributions in the WT contour plots of Co_3_O_4_ as well as the maximum at 7.8 Å^–1^ deriving from Co—Co contributions in the WT contour plots of Co foil (Figure S7, Supporting Information), the intensity maximum at 5.1 Å^–1^ in the WT contour plot of Co‐NG(800) was attributed to Co—N contributions (Figure 2h). Collectively, these results indicate that the Co atoms are isolated (i.e., exist in a single‐atom form) in the aggregates even though the distances between adjacent atoms fall within the range of Co—Co bonds.

Since single atoms have high surface free energy, they are thermodynamically unstable and prone to form clusters or nanoparticles, particularly at elevated temperatures.^[^ ^27^ ^]^ Likewise, aggregates of the Co SACs in the Co‐NG(800) may form via thermally facilitated processes.^[^ ^28^ ^]^ To test this hypothesis, the CoTMPyP‐GO composite was annealed at a lower temperature (400 °C) and the corresponding product was designated as Co‐NG(400). The Co content of Co‐NG(400) was measured by XPS to be 2.55 wt%. HAADF‐STEM confirmed that the Co atoms in the Co‐NG(400) adopted an isolated form and the Co *K*‐edge XAS data indicated that the Co atoms retained the Co–N_4_ coordination structure (Figures S8 and S9 as well as Table S1, Supporting Information). Although the single atoms in the Co‐NG(800) were found to exist in an aggregated form and the distance between adjacent Co atoms were within the van der Waals radii of two Co atoms, Co—Co bonds were not detected. Therefore, each Co atom in the aggregates can still function as an SAC and, due to their proximities, should facilitate interactions with molecules that are of commensurate size, such as polysulfides (vida infra). The aforementioned methodology is advantageous as it overcomes the longstanding challenge associated with high‐temperature annealing processes that often lead to the formation of clusters or particles and thus compromises the catalyst utilization rate.^[^ ^28^ ^]^ The ability to tune the existing forms of the Co SACs on graphene was attributed to the porphyrin complex, which was capable of chelating the Co(II) atoms while forming stabilizing interactions with the underlying graphene substrate. Phthalocyanines, salens, bipyridines, and other heteroaromatics with structures and functions that are similar to those of the porphyrins can be expected to serve as potential alternative organic ligands.

### 2.3 Electrochemical Characterization of Li–S Batteries

Sulfur was impregnated into the aforementioned host materials via a melt‐diffusion method and the resulting composites were designated as S@Co‐NG(800), S@Co‐NG(400), S@NG, and S@G, respectively. The sulfur loading was confirmed by the significant decreases in the specific surface areas and pore values measured for the host materials (Figure S10 and Table S2, Supporting Information). The sulfur contents in these composites were measured to be ≈74 wt% using thermogravimetric analysis (Figure S11, Supporting Information). Coin‐type cells were assembled using the composites as cathodes, Li foil as anodes, and a solution of lithium bis(trifluoromethanesulfonyl)imide (LiTFSI) (1.0 m) in a mixture of 1,3‐dioxolane (DOL) and 1,2‐dimethoxyethane (DME) (1:1 v/v) containing LiNO_3_ (2 wt%) as electrolyte. Preliminary tests demonstrated that the Li–S cells prepared from S@Co‐NG(800) afforded higher performance metrics than the cells prepared from S@Co‐NG(400) (Figure S12, Supporting Information), indicating that the Co SACs exhibited more efficient electrocatalytic effects when present in an aggregated form as opposed to being isolated. Since the cells containing either S@Co‐NG(800) or S@Co‐NG(400) were measured to have similar serial resistance values (i.e., several ohms, Figure S12b, Supporting Information), any impact of differences in electrical conductivity of the host materials on cell performance due to the different annealing temperatures employed can be eliminated. As such, the following section will primarily focus on exploring the electrocatalytic effects displayed by Co‐NG(800) in sulfur cathodes.

**Figure 3:**
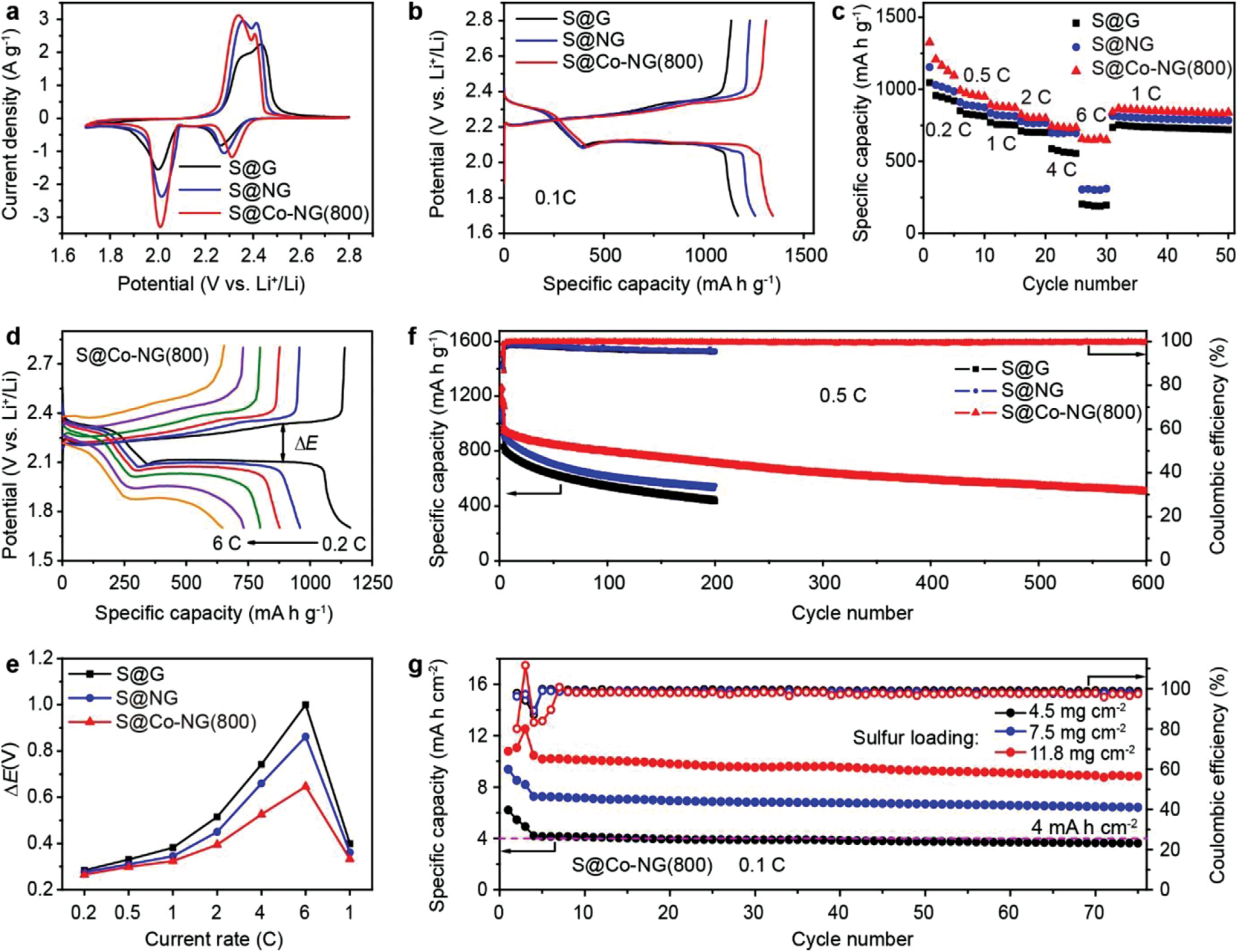
Electrochemical characterization of Li–S cells. a) CV curves recorded at a scan rate of 0.1 mV s^–1^ for different cathodes. b) Charge/discharge profiles recorded at 0.1 C for different cathodes. c) Rate capabilities recorded for different cathodes. d) Charge/discharge profiles recorded at 0.2 C, 0.5 C, 1 C, 2 C, 4 C, and 6 C for the S@Co‐NG(800) cathode. e) Summary of the potential gaps recorded at different current rates for different cathodes. f) Cycling tests recorded at 0.5 C for the cells prepared using different cathodes. g) Cycling tests recorded at 0.1 C for cells prepared using S@Co‐NG(800) at high sulfur loadings. The specific materials analyzed are indicated in the respective legends.

**Figure** **3**a shows a series of cyclic voltammetry (CV) curves recorded for the Li–S cells containing S@Co‐NG(800), S@NG, or S@G at a scan rate of 0.1 mV s^–1^. In the cathodic scan, each curve contains two reduction peaks at ≈2.3 and 2.0 V, which were ascribed to the conversion of S_8_ to soluble lithium polysulfides (Li_2_S*_x_*, 4 ≤ *x* ≤ 8) and then to insoluble Li_2_S_2_ and Li_2_S. In the following anodic scan, the dominate oxidation peak was attributed to the conversion of Li_2_S to S_8_. The conversion of S_8_ to lithium polysulfides at the S@Co‐NG(800) cathode (onset potential of 2.40 V) occurred at more positive potential when compared to the cathodes containing S@NG or S@G (onset potential of 2.37 V). Similarly, oxidation of the Li_2_S at the S@Co‐NG(800) cathode (onset potential of 2.21 V) took place at more negative potential than that recorded for the S@NG and S@G cathodes (onset potential of 2.26 V). Moreover, the S@Co‐NG(800) cathode exhibited the greatest redox peak currents among the three cathodes tested. Galvanostatic charge/discharge profiles were also recorded at a current rate of 0.1 C (1 C = 1675 mA g^–1^). As shown in Figure 3b, the charge and discharge plateaus were consistent with the oxidation and reduction peaks, respectively, that were observed in the CV curves. For comparison, the Li–S cell prepared from the S@Co‐NG(800) composite exhibited a markedly higher specific capacity than the cells that contained either the S@NG or S@G composite (1346 mA h g^–1^ vs 1257 or 1171 mA h g^–1^, respectively). Collectively, these data indicated that the Co SACs embedded in the S@Co‐NG(800) were capable of promoting the cathode reaction kinetics.

The rate capabilities of the cells were evaluated by increasing the current rate from 0.2 C to 6 C (Figure 3c). Of the three different cathodes tested (i.e., S@Co‐NG(800), S@NG, and S@G), the cathodes containing the S@Co‐NG(800) consistently exhibited the highest specific capacity. In particular, at 6 C, the specific capacity measured for the S@Co‐NG(800) cathode was significantly higher than the values measured for the S@NG and S@G cathodes (648 mA h g^–1^ vs 301 and 190 mA h g^–1^, respectively). When the current rate was re‐set back to 1 C, a specific capacity of 864 mA h g^–1^ was measured for the S@Co‐NG(800) cathode. The value was comparable to the initial value of 877 mA h g^–1^, indicating good reversibility of the S@Co‐NG(800) cathode. Indeed, after 50 subsequent cycles, a specific capacity of 838 mA h g^–1^ was measured, corresponding to a capacity retention of 97%. The excellent rate capability and capacity retention of the S@Co‐NG(800) cathode were attributed to the embedded Co SACs that promote the cathode reactions of Li–S cells. Figure 3d shows a series of galvanostatic charge/discharge profiles recorded at current rates that ranged from 0.2 C to 6 C for the S@Co‐NG(800) cathode, where the potential difference (Δ*E*) between the charge and discharge plateau was correlated with the polarization of the cathode.^[^ ^22d^ ^]^ In contrast to the galvanostatic profiles acquired for the S@NG and S@G cathodes (Figure S13, Supporting Information), the S@Co‐NG(800) cathode was capable of maintaining the charge/discharge plateau as the current rate was increased. As shown in Figure 3e, the S@Co‐NG(800) cathode consistently exhibited a smaller Δ*E* than the S@NG or S@G cathodes and the difference was magnified at high current rates (e.g., 0.65 V vs 0.86 or 1.00 V, respectively, at 6 C). The smaller polarization recorded for the S@Co‐NG(800) cathode reflected faster reaction kinetics and was attributed the electrocatalytic effects provided by the Co SACs.

The long‐term cycling stabilities of the Li–S cells were measured at 0.5 C since the shuttle effect of polysulfides is pronounced at low current rates (Figure 3f). The S@Co‐NG(800) cathode delivered an initial specific capacity of 972 mA h g^–1^ and retained a value of 505 mA h g^–1^ after 600 cycles, corresponding to a capacity decay of only 0.08% per cycle. For comparison, the capacity decays were measured to be 0.21% and 0.24% per cycle for the S@NG and S@G cathodes, respectively, after 200 charge/discharge cycles. Additionally, the S@Co‐NG(800) cathode also exhibited a constant Coulombic efficiency that was close to 100% over the long‐term cycling study, which is in sharp contrast to the significant decreases observed for the S@NG and S@G cathodes.

To further test the superlative performance exhibited by S@Co‐NG(800), Li–S cells were prepared with cathodes that contained relatively high sulfur loadings. As summarized in Figure 3g, at sulfur loadings of 4.5, 7.5, or 11.8 mg cm^–2^, the corresponding Li–S cells exhibited ultrahigh area specific capacities of 6.20, 9.37, or 12.52 mA h cm^–2^ at 0.05 C, respectively. The values are significantly higher than the threshold required for commercial lithium‐ion batteries (4 mA h cm^–2^).^[^ ^29^ ^]^ Cycling stability tests were further performed at 0.1 C and the data showed that the Li–S batteries prepared with gradually increased sulfur loadings worked well along with Coulombic efficiencies that were close to 100%. In particular, the Li–S cell prepared at a sulfur loading of 11.8 mg cm^–2^ displayed an area specific capacity of 10.47 mA h cm^–2^ initially and 8.86 mA h cm^–2^ after 75 cycles at 0.1 C, corresponding to a capacity retention of 85%. The area sulfur loading and area specific capacity values described are higher than the values reported for the Li–S batteries that contained SACs (Table S3, Supporting Information),^[^ ^22^, ^30^ ^]^ highlighting the advantages of using SAC aggregates to promote the electrochemical performance of sulfur cathodes.

### 2.4 Characterization of the Electrocatalytic Effect

**Figure 4:**
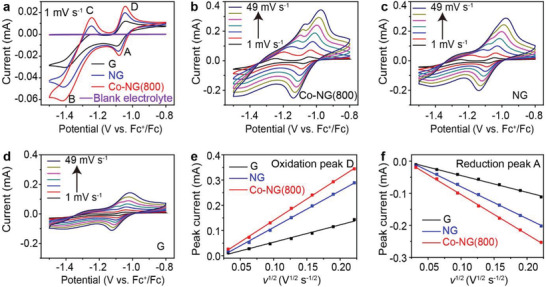
Electrocatalytic characterization using a three‐electrode system. a) CV curves recorded at a scan rate of 1 mV s^–1^ for Co‐NG(800), NG, and G electrodes. b–d) CV curves recorded at a series of scan rates of 1, 4, 9, 16, 25, 36, and 49 mV s^–1^ for electrodes containing b) Co‐NG(800), c) NG, or d) G. e,f) Plots of *I*_p_ values, derived from the oxidation peak D or the reduction peak A, as function of *v* ^1/2^ for the different electrodes. The specific materials analyzed are indicated in the respective legends.

The electrocatalytic properties of Co‐NG(800) were further investigated using a three‐electrode system (see the Experimental Section for details). **Figure** **4**a compares a series of CV curves that were recorded at a scan rate of 1 mV s^–1^ for electrodes containing Co‐NG(800), NG, or G. As opposed to a featureless CV curve that was recorded from a blank electrolyte (i.e., no added Li_2_S_6_), the two reduction peaks detected at ≈ –1.08 and –1.41 V in the cathodic scan (labeled as A and B) were ascribed to the conversion of S_8_ to polysulfides and then to Li_2_S, respectively. In the anodic scan, two oxidation peaks were detected at –1.24 and –1.04 V (labeled as C and D) and were attributed to the reverse reactions (i.e., Li_2_S to polysulfides and then to S_8_, respectively). The intensities of the peak currents of the redox events followed the order: Co‐NG(800) > NG > G. This finding indicated that the SACs present in the Co‐NG(800) facilitated the conversions of sulfur at the corresponding electrode. Furthermore, as shown in Figure S14 (Supporting Information), aggregates of the Co SACs exhibited a larger electrocatalytic effect when compared the analogs that remained in an isolated state.

To gain a deeper understanding of the underlying reaction kinetics, CV curves were collected at a series of scan rates that ranged from 1 to 49 mV s^–1^. As shown in Figure 4b–d, the Co‐NG(800) electrode consistently exhibited the largest redox peak currents at each scan rate among the different electrodes, which indicated the fastest reaction kinetics on the Co‐NG(800) electrode and were attributed to the electrocatalytic effect of the single‐atom Co catalyst. The reaction kinetics was quantitatively assessed by measuring the corresponding Li‐ion diffusion coefficient (*D*_Li_^+^) values using the Randles–Sevcik equation (Equation (1))

(1) $$I_{P}=2.69\times 10^{5}n^{3/2}AD_{{\mathrm{Li}}^{+}}^{1/2}C_{{\mathrm{Li}}^{+}}\nu^{1/2}$$

where *I*_p_ is the peak current of the redox reaction, *n* is the number of electrons involved in the electrochemical reaction (*n* = 2 for Li–S batteries), *A* is the area of the electrode, *C*_Li_^+^ is the concentration of Li ions in the electrolyte, and *ν* is the scan rate.

The peaks labeled as A and D were used to calculate the *D*_Li_^+^ values since they maintained their shapes as the scan rate was increased. As shown in Figure 4e,f, the *I*_p_ increased linearly as a function of the square root of scan rate (*v* ^1/2^) in both oxidation and reduction processes (reflective of a mass transport limited process), from which the *D*_Li_^+^ values were derived. During oxidation, the *D*_Li_^+^ value measured for the Co‐NG(800) electrode (i.e., 5.13 × 10^–10^ cm^2^ s^–1^) was 1.4 and 6.1 times higher than the values measured for the NG and G electrodes (i.e., 3.73 × 10^–10^ and 8.42 × 10^–11^ cm^2^ s^–1^, respectively). Similarly, for the reduction reaction, the *D*_Li_^+^ value measured for the Co‐NG(800) electrode (i.e., 2.72 × 10^–10^ cm^2^ s^–1^) was 1.6 and 5.6 times higher than those measured for the other electrodes (i.e., 1.75 × 10^–10^ and 4.87 × 10^–11^ cm^2^ s^–1^, respectively). Notably, the Co‐NG(800) electrode (i.e., the SACs that exist in an aggregated form) exhibited faster reaction kinetics when compared with the Co‐NG(400) electrode (i.e., the Co SACs that exist in an isolated form) (Figure S14, Supporting Information). Likewise, the *D*_Li_^+^ values that corresponded to the peaks A and D for the Co‐NG(800) electrode were measured to be 2.1 and 2.2 times higher than the values measured for the Co‐NG(400) electrode (Table S4, Supporting Information).

**Figure 5:**
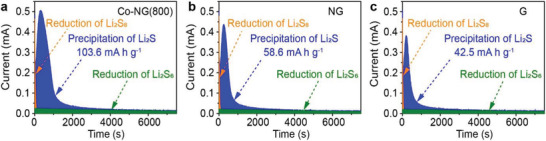
Potentiostatic Li_2_S precipitation recorded for different electrodes. a) Co‐NG(800) electrode. b) NG electrode. c) G electrode.

The conversion of polysulfides to Li_2_S is a key step that determines the specific capacities of Li–S cells. To investigate the electrocatalytic effect of the single‐atom Co catalyst on the conversion of polysulfides to Li_2_S, a series of Li_2_S precipitation experiments were performed using the different electrodes prepared from Co‐NG(800), NG, or G (see the Experimental Section). As shown in **Figure** **5**, each potentiostatic discharge profile was consisted of a sharp decrease (colored orange), a dominant bump (blue), and a slowly decreasing baseline (green), which could be attributed to the reduction of residual Li_2_S_8_, the nucleation and growth of Li_2_S, and the reduction of Li_2_S_6_, respectively.^[^ ^31^ ^]^ As expected, the capacity corresponding to the Li_2_S precipitation recorded for the Co‐NG(800) cathode (103.6 mA h g^–1^, normalized to mass of sulfur) was significantly higher than those recorded for the NG and G cathodes (58.6 and 42.5 mA h g^–1^, respectively). This result indicated that the Co SAC aggregates substantially promoted the conversion of Li_2_S. Aggregates of the Co SACs were also found to promote Li_2_S precipitation more readily than their isolated form, as the corresponding specific capacity recorded for the former (103.6 mA h g^–1^) was 1.5 times higher than that recorded for the latter (68.8 mA h g^–1^, Figure S15, Supporting Information).

### 2.5 Elucidation of the Electrocatalytic Mechanism

**Figure 6:**
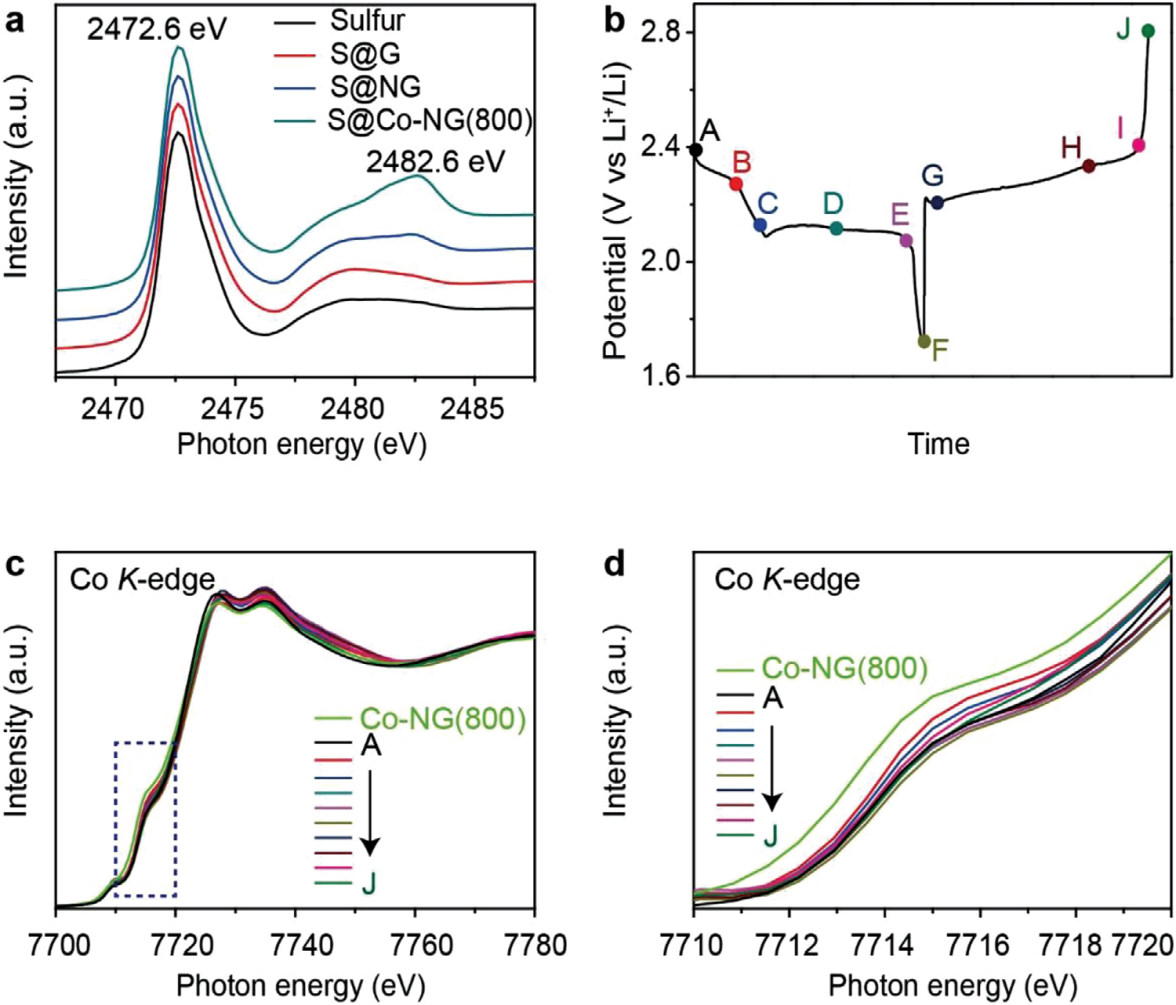
Electrocatalytic characterization by XANES spectroscopy. a) S *K*‐edge XANES spectra of pristine sulfur, S@G, S@NG, and S@Co‐NG(800) (indicated). b) Charge/discharge curves showing a series of electrochemical states for collecting Co *K*‐edge XANES spectra: A, the initial state; B, discharged to 2.25 V; C, discharged to 2.1 V; D, discharged to the middle of the second plateau; E, discharged to 2.05 V; F, discharged to 1.7 V; G, charged to the beginning of the first plateau; H, charged to the beginning of the second plateau; I, charged to 2.39 V; J, charged to 2.8 V. c) Co *K*‐edge XANES spectra collected at the different electrochemical states for the S@Co‐NG(800) cathode (indicated). d) Magnified profiles of the pre‐edge region of Co *K*‐edge XANES spectra (indicated).

To unveil the electrocatalytic mechanism of the Co‐NG(800), XANES spectroscopy was used to analyze the interactions formed between Co‐NG(800) and the sulfur species present during the charge/discharge processes. **Figure** **6**a shows the S *K*‐edge XANES spectra of pristine sulfur as well as the sulfur that was infiltrated into the different host materials. The S *K*‐edge XANES spectrum of pristine sulfur featured an intense absorption‐edge peak at 2472.6 eV and a broad post‐edge absorption at ≈2480.0 eV. There was no noticeable change in the XANES spectrum recorded for the S@G composite, indicating that the interactions formed between sulfur and G were negligible. By contrast, a new absorption peak was detected at 2482.6 eV for the S@Co‐NG(800) and S@NG composites. The difference was attributed to the formation of new interactions, presumably between the sulfur and the N heteroatoms and/or the Co SACs, that facilitate the cathode reactions of Li–S batteries. Significantly, the new absorption peak recorded for S@Co‐NG(800) was stronger than that recorded for S@NG, indicating that the Co SACs provide stronger interactions and thus may play a dominate role in promoting the sulfur cathode reactions.

Co *K*‐edge XANES spectra were collected from the cathode materials retrieved at different charge/discharge states (as shown in Figure 6b) and compared with data recorded for pristine Co‐NG(800). After sulfur infiltration, absorption edge of the Co *K*‐edge XANES spectra was found to shift to a higher energy region by ≈1 eV (see black line vs green line, Figure 6c,d), a finding that is consistent with the S *K*‐edge spectra and indicates the enhancement of the oxidation state of the single‐atom Co due to the sulfur coordination. Over the charge/discharge cycle, the absorption edge remained consistently at a higher region along with largely unchanged spectroscopic features (i.e., pre‐edge feature, shoulder peak, and absorption‐edge peak). These results indicated that the structure of the single‐atom Co aggregates was essentially unchanged during the charge/discharge cycle and that interactions formed between the Co SACs and the sulfur species persisted throughout the electrochemical process.

To further clarify the electrocatalytic effect of the single‐atom Co aggregates, a series of theoretical calculations were performed by DFT with the D3 empirical dispersion effect correction, which was previously used in the studies of Li–S batteries.^[^ ^22a^ ^]^ As shown in Figure S16 (Supporting Information), three representative substrate models were built using the coordination environment of the Co SACs (i.e., Co–N_4_). After geometry optimization, the distances between the adjacent Co atoms were determined to be 0.32, 0.40, and 1.37 nm, consistent with experimental values (Figure S6, Supporting Information). The models were designated as Co‐NG‐0.32, Co‐NG‐0.40, and Co‐NG‐1.37, respectively.

**Figure 7:**
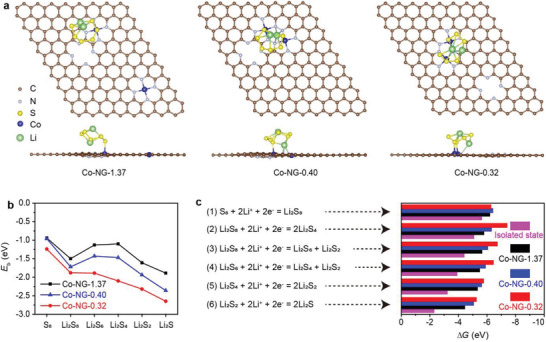
DFT data for analyzing the adsorption and electrocatalytic effects. a) Top and side views of the most stable adsorption configurations of a Li_2_S_6_ molecule on Co‐NG‐1.37, Co‐NG‐0.40, or Co‐NG‐0.32. b) Calculated *E*_b_ values for various sulfur species on the different substrates (indicated). c) Sequential reactions in a discharge process and the corresponding Δ*G* values as calculated on the different substrates and at an isolated state (indicated).

**Figure** **7**a shows the adsorption of a Li_2_S_6_ molecule on each of the different substrate models in their most stable forms. In the Co‐NG‐1.37 model, the distance between the two Co atoms (1.37 nm) is beyond the size of a Li_2_S_6_ molecule and thus the absorption can occur only through one Co atom. By contrast, in the Co‐NG‐0.40 and Co‐NG‐0.32 models, a Li_2_S_6_ molecule can be simultaneously absorbed by two Co atoms because the distances of the adjacent Co atoms (i.e., 0.40 and 0.32 nm, respectively) are commensurate with the size of a Li_2_S_6_ molecule. Therefore, the bidentate nature of such absorption can be expected to enhance the binding with a Li_2_S_6_ molecule. The adsorption of other sulfur species was found to be similar to the manners described for Li_2_S_6_ on the different substrates (see Figures S17–S19, Supporting Information, for details). The N heteroatoms present in the graphene plane were found to interact with the Li ions in all three models. Figure 7b summarizes the binding energy (*E*_b_) values calculated for each model, which follow the order: Co‐NG‐0.32 > Co‐NG‐0.40 > Co‐NG‐1.37. These results indicate that the aggregates of Co SACs exhibit stronger absorption characteristics with various sulfur species than their isolated forms.

Figure 7c summarizes a series of sulfur cathode reactions in a discharge process and the changes in the Gibbs free energy (Δ*G*) values calculated for the reactions on the different substrates. The Δ*G* values calculated for the conversion of Li_2_S_2_ to Li_2_S is the least negative, indicating that the reaction is the rate‐limiting step.^[^ ^12^, ^22^ ^]^ The Δ*G* values calculated for this step using the Co‐NG‐0.32 and Co‐NG‐0.40 models are more negative than those calculated with the Co‐NG‐1.37 model or an isolated state. This result is consistent with the experimental data which showed that the single‐atom Co aggregates facilitate the conversion of Li_2_S_2_ to Li_2_S more efficiently than their isolated form. Furthermore, the Δ*G* values for each step were found to change in the following order: Co‐NG‐0.32 > Co‐NG‐0.40 > Co‐NG‐1.37 > isolated state, suggesting that the single‐atom Co aggregates promote the entire discharge process more markedly than their isolated form. Collectively, these results explain why the Li–S cells prepared using the Co‐NG(800) material as a sulfur host exhibit better performance in terms of specific capacity, rate capability, and cycling stability when compared with the analogous cells that were prepared from the Co‐NG(400) material.

## 3 Conclusion

In summary, aggregates of Co SACs on graphene were synthesized from readily available reagents, including a Co(II) porphyrin complex and GO. The Co atoms on the graphene sheets can migrate and aggregate when heated yet, due to the intrinsic coordination environment of the metals, are prevented from forming Co—Co bonds in the aggregates. DFT calculations revealed that the Co atoms can form synergistic interactions with relatively large reactant sulfur species and, as a result, maximize electrocatalytic effects. A series of three‐electrode measurements and Li_2_S precipitation tests revealed that electrodes containing the Co SAC aggregates exhibited faster Li^+^ diffusion coefficients and an increased Li_2_S precipitation when compared to control electrodes that contained the Co SACs existing in an isolated form. The Li‒S cells prepared using Co‐NG(800) as a sulfur host also showed outstanding performance metrics, including a high specific capacity (1346 mA h g^‒1^ at 0.1 C), a high rate capacity (648 mA h g^‒1^ at 6 C), and excellent cycling stability (505 mA h g^−1^ at 0.5 C after 600 cycles, corresponding to a decay of only 0.08% per cycle). Moreover, an ultrahigh area capacity of 12.52 mA h cm^−2^ was measured from a Li–S cell with a high sulfur loading of 11.8 mg cm^−2^. Collectively, these results provide new insights into the electrocatalytic processes in the sulfur cathodes of Li‒S cells, and the methodology described offers a general strategy for adapting other SACs for use in contemporary electrocatalytic reactions.

## 4 Experimental Section

The details of the methods are provided in the Supporting Information.

## Conflict of Interest

The authors declare no conflict of interest.

## Supporting information

Supporting Information

## Data Availability

Research data are not shared.
