# Supplementary material for: Single‐Atom Catalyst Aggregates: Size‐Matching is Critical to Electrocatalytic Performance in Sulfur Cathodes
Source: Adv Sci (Weinh). 2021 Nov 16;9(3):2103773. doi: 10.1002/advs.202103773 (PMC8787415; doi:10.1002/advs.202103773)
Supplement: Supplementary file 1 — Supporting Information [file ADVS-9-2103773-s001.pdf]

WILEY-VCH

© 2021 Wiley-VCH GmbH

ADVANCED SCIENCE
Open Access

Supporting Information

for *Adv. Sci.,* DOI: 10.1002/advs.202103773

Single-Atom Catalyst Aggregates: Size-Matching is Critical to Electrocatalytic Performance in Sulfur Cathodes

Xiaodong Meng, Xing Liu, Xueying Fan, Xin Chen, Shang Chen, Yongqiang Meng, Manyun Wang, Ji Zhou, Song Hong, Lei Zheng, Guosheng Shi, Christopher W. Bielawski, Jianxin Geng**

Supporting Information

Single-Atom Catalyst Aggregates: Size-Matching is Critical to Electrocatalytic Performance in Sulfur Cathodes

Xiaodong Meng, Xing Liu, Xueying Fan, Xin Chen, Shang Chen, Yongqiang Meng, Manyun Wang, Ji Zhou, Song Hong, Lei Zheng, Guosheng Shi, Christopher W. Bielawski, Jianxin Geng**

Methods

Materials. Pyrrole was purchased from Aladdin Co., Ltd. 4-Pyridinecarboxaldehyde was purchased from Adamas-beta reagent Co., Ltd. Sublimed sulfur (>99%) was purchased from Xilong Chemical Co., Ltd. Li_2S was purchased from Sigma-Aldrich (Shanghai) Co., Ltd. All other reagents and solvents were purchased from Sinopharm Chemical Co., Ltd., and used without any further purification. 5,10,15,20-meso-tetrakis(N-methyl-4-pyridinyl)porphyrin tetraiodide ([TMPyP]I_4) and its cobalt(II) derivative [CoTMPyP]I_4 were synthesized according to previous reports (Figure S1, Supporting Information).[1] All reactions were carried out under an argon atmosphere. Graphene oxide (GO) was synthesized by the modified Hummers' method.[2]

Synthesis of the Co single-atom catalysts (SACs) on graphene. The Co SACs were prepared in three steps: attachment of the Co(II) porphyrin complex to GO, hydrothermal reduction of the composite, and thermal treatment. An aqueous solution of [CoTMPyP]I_4 (2.5 mg mL^{-1}, 30 mL) was first added into an aqueous suspension of GO (5 mg mL^{-1}, 30 mL) under mild stirring. A precipitate was observed and labeled as CoTMPyP-GO. The precipitate was separated by filtration and washed with deionized water until the filtrate became colorless. The composite was re-dispersed in deionized water (30 mL) and hydrazine hydrate (99%, 1.5 mL) was added to the aqueous suspension with stirring. The mixture was then transferred to a Teflon-lined autoclave (50 mL) and then subjected to hydrothermal treatment at 180 °C for 20 h. The resulting material was washed with deionized water and freeze dried. Finally, the

obtained material was thermally heated using a tube furnace at 800 °C or 400 °C for 2 h in argon atmosphere under a gas flow of 500 sccm. The products were designated as Co-NG(800) or Co-NG(400), respectively. As controls, NG was prepared by replacing [CoTMPyP]I_4 with [TMPyP]I_4 and G was prepared in analogous manner but without the addition of the porphyrin.

Characterization. Zeta potential data were collected on a Malvern Zetasizer Nano ZS90 with three parallel experiments. The Zeta potential value was determined from the average value for each sample. UV-vis spectra were recorded on a Shimadzu UV 2600 spectrophotometer. The samples used in zeta potential and UV-vis spectroscopy measurements were prepared by mixing different volumes of a GO suspension (0.5 mg mL^{-1}) and a porphyrin solution (0.5 mg mL^{-1}) to obtain targeted ratios of the two components, followed by sonication for 5 min. X-ray diffraction (XRD) data were recorded on a Bruker D2 PHASER X-ray diffractometer (Cu $K\alpha$, λ = 0.154 nm). X-ray photoelectron spectroscopy data were obtained on an ESCALAB 250Xi X-ray photoelectron spectrometer using a monochromated Al $K\alpha$ radiation (1486.7 eV). The N contents of the samples were measured using organic elemental analyzer vario EL cube produced by Elementar Analysensysteme GmbH. N_2 adsorption/desorption measurements were performed on a Micromeritics ASAP 2460 Surface Area and Porosity Analyzer at 77K. The specific surface area and pore volume values were obtained using the Brunauer−Emmett−Teller (BET) method and the Barrett−Joyner−Halenda (BJH) method, respectively. Transmission electron microscope images and elemental mapping images were obtained on a JEOL JEM-2100F microscope. Aberration corrected high-angle annular dark field scanning transmission electron microscopy (HAADF-STEM) measurements were conducted on JEOL JEM-ARM200F microscope operating with an acceleration voltage of 200 kV and equipped with single spherical aberration correctors. Thermogravimetric analysis

(TGA) data were collected on a Netzsch TG 209 F3 at a heating rate of 10 °C min^{-1} from 40 °C to 800 °C under an atmosphere of argon or air.

The S *K*-edge X-ray absorption near-edge structure (XANES) spectra and Co *K*-edge X-ray absorption spectroscopy (XAS) data were collected on the tender X-ray beamline (4B7A) and hard X-ray beamline (1W1B and 1W2B) of the Beijing Synchrotron Radiation Facility (BSRF), respectively. The as-prepared cathode electrodes containing S@Co-NG(800), S@NG, S@G, and pristine sulfur were used for collecting S *K*-edge XANES spectra. The S *K*-edge XANES spectra were collected in total electron yield mode. The Co *K*-edge XAS data were collected from Co-NG(800) powder and the cathode materials that were retrieved from the Li–S cells at specific charge/discharge states. Before measurement, the cathode materials were washed with tetrahydrofuran. The Co *K*-edge XAS data were collected in the fluorescence mode. The acquired XAS data were analyzed by using ATHENA and ARTEMIS modules in IFEFFIT software package according to the standard data analysis procedures.

Electrochemical measurements. Three-electrode tests were performed in an argon-filled glove box. A Li_2S_6 solution was prepared by dispersing sublimed S (160.3 mg, 5 mmol) and Li_2S (46.1 mg, 1 mmol) in a solution of lithium bis(trifluoromethanesulfonyl)imide (LiTFSI, 1.0 M, 100 mL) in a mixture of 1,3-dioxolane (DOL) and 1,2-dimethoxyethane (DME) (1 : 1 in v/v) that contained a $LiNO_3$ additive (2.0 wt%). The Li_2S_6 solution (15 mL) was used as an electrolyte in the three-electrode tests. The Co-NG(800), Co-NG(400), NG, or G material was mixed with polyvinylidene fluoride (PVDF) at a mass ratio of 8 : 2 in *N*,*N*-dimethylformamide (DMF) and stirred to obtain homogeneous slurries. Glassy carbon electrodes were coated with the obtained slurries with the active material being ca. 10 μg and, after being dried in a vacuum oven at 40 °C for 6 h, used as working electrodes. Li foil and a ferrocenium/ferrocene (Fc^+/Fc) electrode were used as the counter and reference electrodes, respectively. The Fc^+/Fc electrode was prepared using ferrocenium hexafluorophosphate (33.1

mg, 0.1 mmol) and ferrocene (18.6 mg, 0.1 mmol) as standard redox couples, anhydrous *N*-methyl-2-pyrrolidone (1 mL) as the solvent, and a Pt wire. The potential of the Fc^+/Fc electrode was measured at 0.66 V versus standard hydrogen electrode. Cyclic voltammetry measurements were performed with a CHI 760E electrochemical workstation from −0.8 to −1.5 V at different scan rates from 1 to 49 mV s^{-1}.

Cathode slurries were first prepared by mixing S@Co-NG(800), S@Co-NG(400), S@NG, or S@G composites with carbon black and PVDF binder at a mass ratio of 80 : 15 : 5 in DMF. Cathode electrodes were prepared by coating each of the slurries on carbon-coated aluminum foils and dried in a vacuum oven at 40 °C for 12 h. Sulfur loadings were controlled at a range of 0.5–1 mg cm^{-2} and, for the S@Co-NG(800) cathode, with sulfur loadings of 4–12 mg cm^{-2} were also prepared. Li foil was used as the counter electrode and separated from the cathode by Celgard 3501 membrane. A LiTFSI solution (1.0 M) in a mixture of DOL and DME (1 : 1 in v/v) with $LiNO_3$ additive (2.0 wt%) was used as electrolyte. CR2025 type Li−S cells were assembled in an argon-filled glove box. For the cathodes with 0.5−1 mg cm^{-2} and 4–12 mg cm^{-2} sulfur loadings, electrolyte/sulfur ratios were 30 μL mg^{-1} and 10 μL mg^{-1}, respectively. The cells were tested on a LAND 2100A battery test system at room temperature. Cyclic voltammetry (CV) measurements were performed with CHI 760E electrochemical workstation from 1.7 to 2.8 V at a scan rate of 0.1 mV s^{-1}.

Potentiostatic Li_2S precipitation experiments were performed using CR2025 type coin cells. Cathode electrodes were prepared by coating a slurry that contained Co-NG(800), Co-NG(400), NG, or G and PVDF as binder (8 : 2 in m/m) in DMF on carbon-coated aluminum foils, followed by drying in a vacuum oven at 40 °C for 12 h. The mass of each material on the cathode was ca. 0.28 mg. Li foil was used as the anode. The coin cells were assembled in argon-filled glove box. A Li_2S_6-containing electrolyte (0.2 M, 25 μL), which was prepared by dispersing sublimed S (320.6 mg, 10 mmol) and Li_2S (92.2 mg, 2 mmol) in the electrolyte (10

mL) used for assembling Li–S cells, was loaded on the cathode side and an electrolyte without Li_2S_6 (25 μL) was loaded on the anode side. The cells were first galvanostatically discharged to 2.09 V at 0.1 C, then potentiostatically discharged at 2.05 V until the current decreased to 0.01 mA. The specific capacities corresponding to Li_2S deposition on the cathode were evaluated following Faraday's law.

DFT calculations. All the spin-polarized calculations were performed using density functional theory (DFT) method implemented in the Vienna ab initio simulation package (VASP)[3]. The projector augmented wave (PAW) method was used to describe electron ion interactions. The generalized gradient approximation (GGA) in the form of Perdew-Burke-Ernzerh function was adopted to describe electron exchange and correlation energy.[3-4] According to previous calculations of the electrochemist in sulfur cathodes,[5] the cutoff energy for plane-wave basis was set as 400 eV and the total energy convergence was set to be lower than 10^{-5} eV, with the force convergence set at 0.01 eV $Å^{-1}$ for geometry optimization. We also increased the cutoff energy to 520 eV to obtain the binding energy between the Li_2S_6 and Co-NG-0.32 (−1.88 eV), a result that is in line with value obtained with a cutoff energy of 400 eV (−1.89 eV) and thus indicate the cutoff energy of 400 eV is suitable in this research. The Gaussian smearing of 0.05 eV and 1 × 1 × 1 Γ-centered Monkhorst-Pack were used for geometry optimization. The DFT-D3 empirical correction method was employed to describe the dispersion effect and van der Waals interactions.[6] A supercell of graphene containing 11 × 10 unit cells was used to model Co-NG systems. Three substrates with different distribution of Co atoms were built. After geometry optimization, the Co-Co distance was 0.32 nm, 0.40 nm, and 1.37 nm (Figure S16, Supporting Information) and the corresponding average Co-N distance was 1.88 Å, 1.89 Å, 1.87 Å, respectively. These models were in agreement with experimental results.

The binding energy (E_b) between the sulfur species and the three different substrates was calculated using the following equation (equation 1):

$E_b = E_{total} - E_{adsorbate} - E_{substrate}$ (1)

where E_{total}, $E_{adsorbate}$ and $E_{substrate}$ are the total energies of adsorption system, adsorbates and substrates, respectively. And the calculations were done at 0 K without ZPE calibrations.

The changes in the Gibbs free energy (ΔG) in the electrochemical processes of Li−S battery was calculated as follows (equation 2):

$\Delta G = \Sigma G_{product} - \Sigma G_{reactant}$ (2)

where $G_{product}$ and $G_{reactant}$ are the Gibbs free energies of the products and reactants and the contributions of entropy and volume effects are negligible.[7]

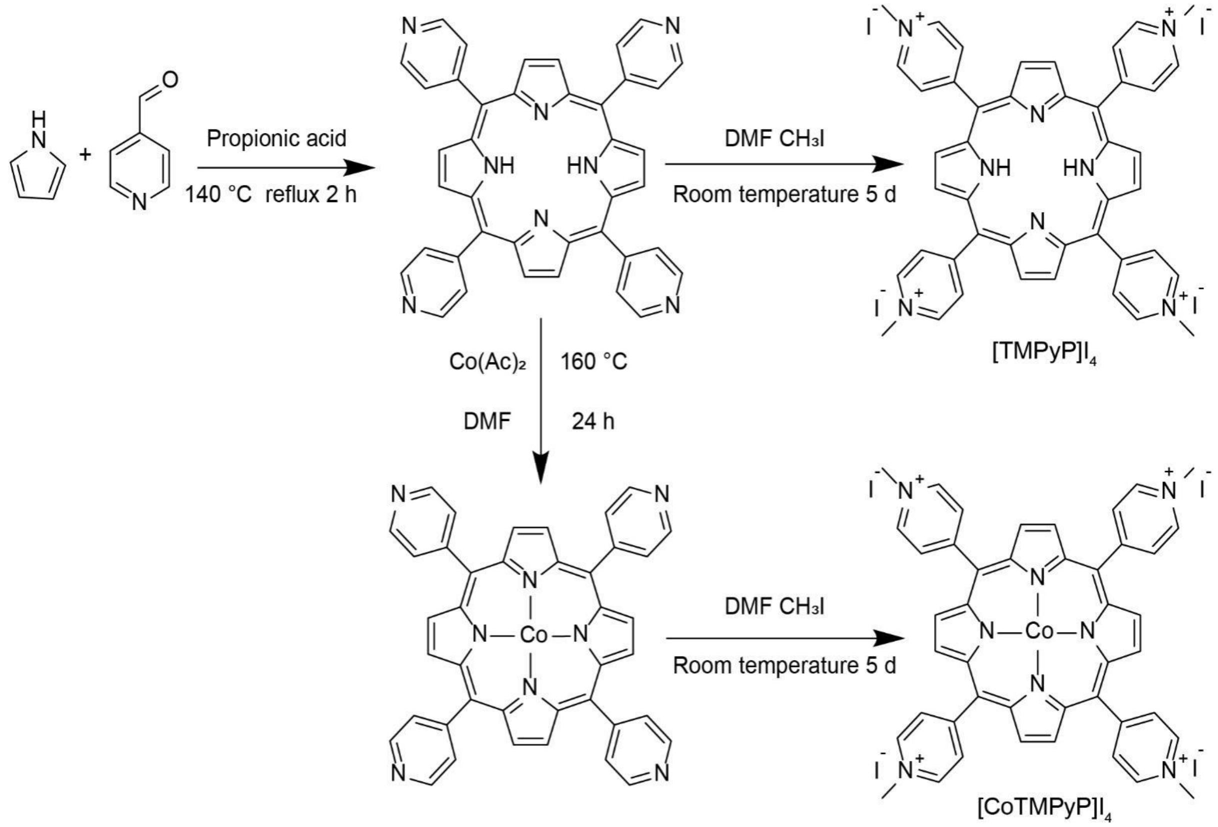

Figure S1. Synthetic route used to prepare $[TMPyP]I_4$ and $[CoTMPyP]I_4$.

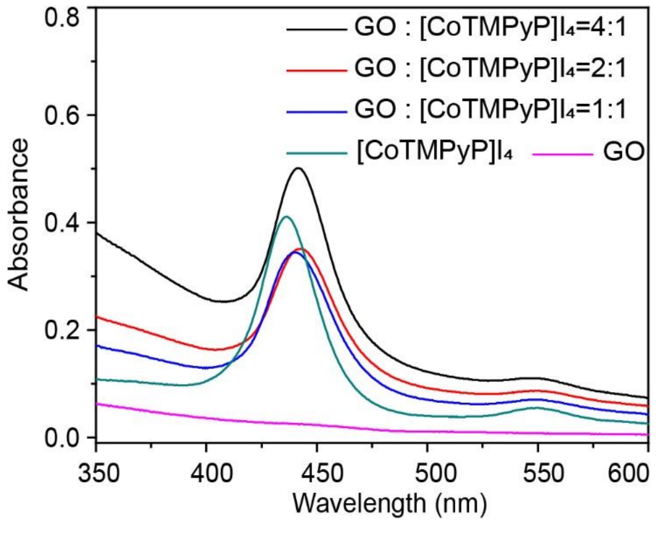

Figure S2. UV-vis spectra recorded for various CoTMPyP-GO composite suspensions at different ratios of GO to $[CoTMPyP]I_4$ (indicated).

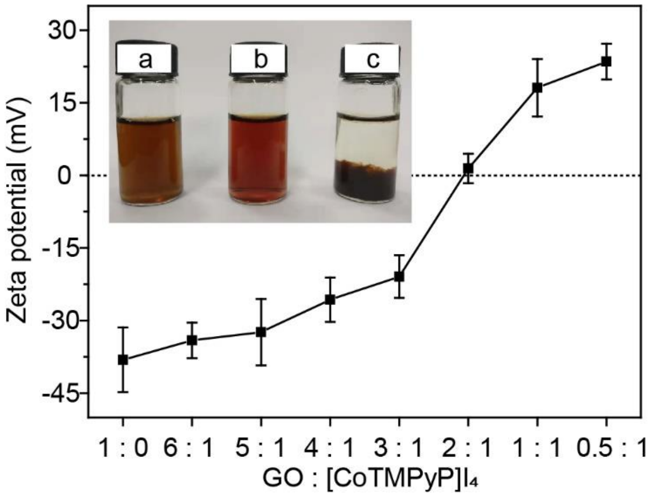

Figure S3. Zeta potential of the CoTMPyP-GO composite suspensions as function of the ratio of GO to [CoTMPyP]I_4. The inset showed the digital photos of the GO suspension (a), the [CoTMPyP]I_4 solution (b), and the composite prepared at the component ratio of 2 : 1 (c).

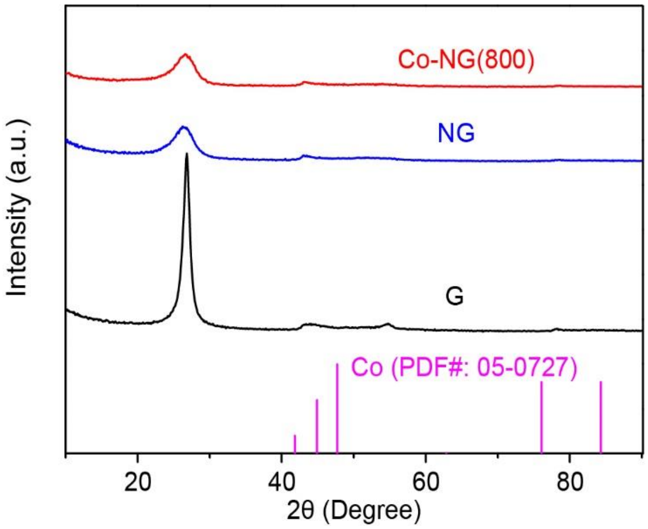

Figure S4. XRD patterns measured for Co-NG(800), NG, and G. The XRD diffractions of metallic Co are shown as vertical lines.

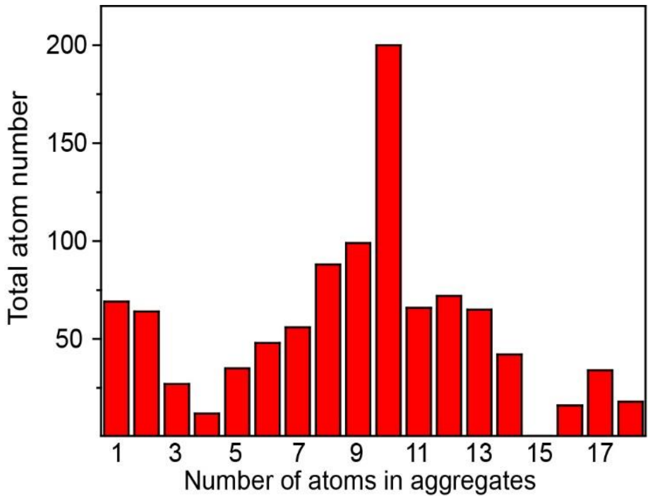

Figure S5. Statistical distribution of the number of Co single atoms in the aggregates.

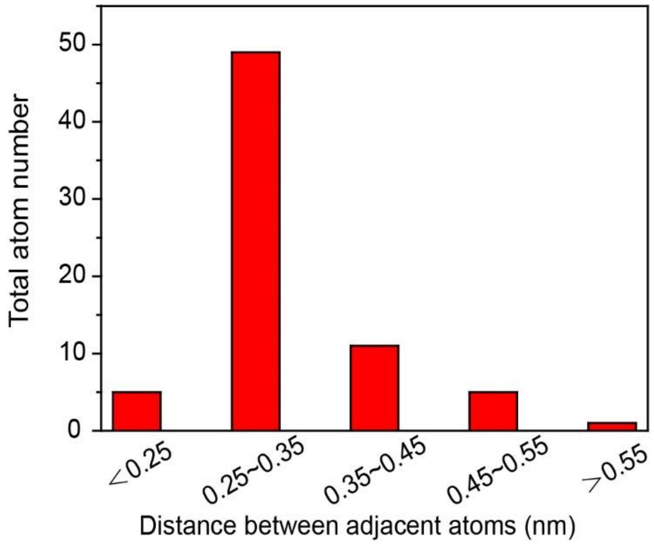

Figure S6. Statistical distribution of the distance between adjacent Co atoms in the aggregates.

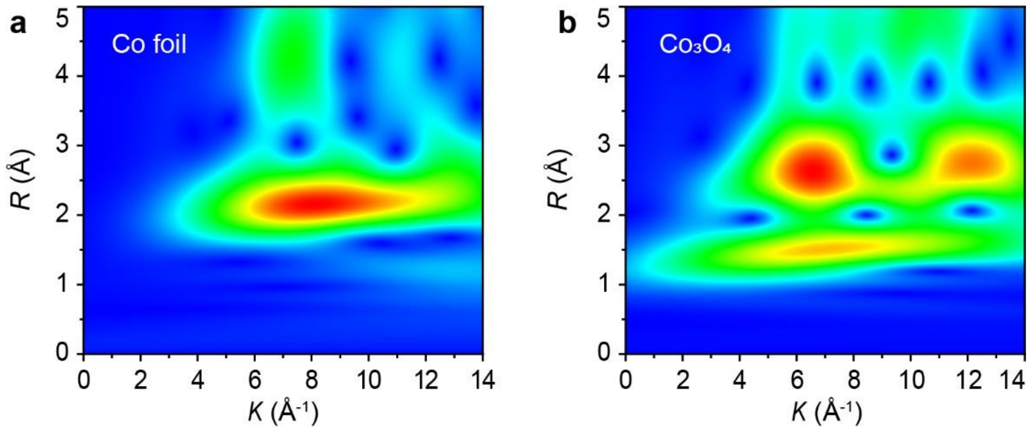

Figure S7. WT contour plots of the EXAFS data for: a) Co foil and b) Co_3O_4.

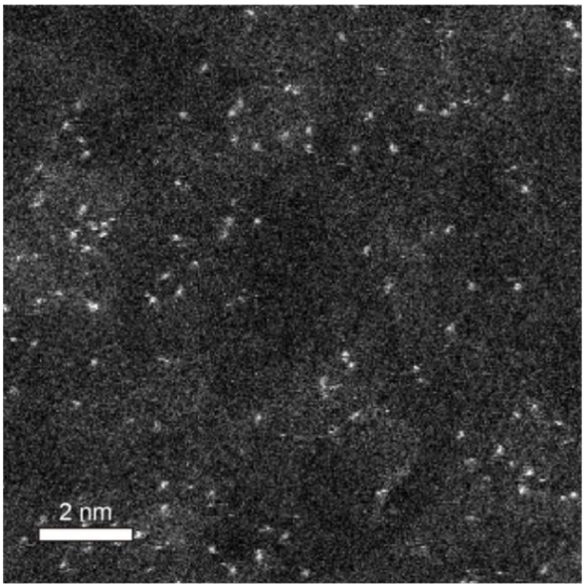

Figure S8. HAADF-STEM image of the Co-NG(400).

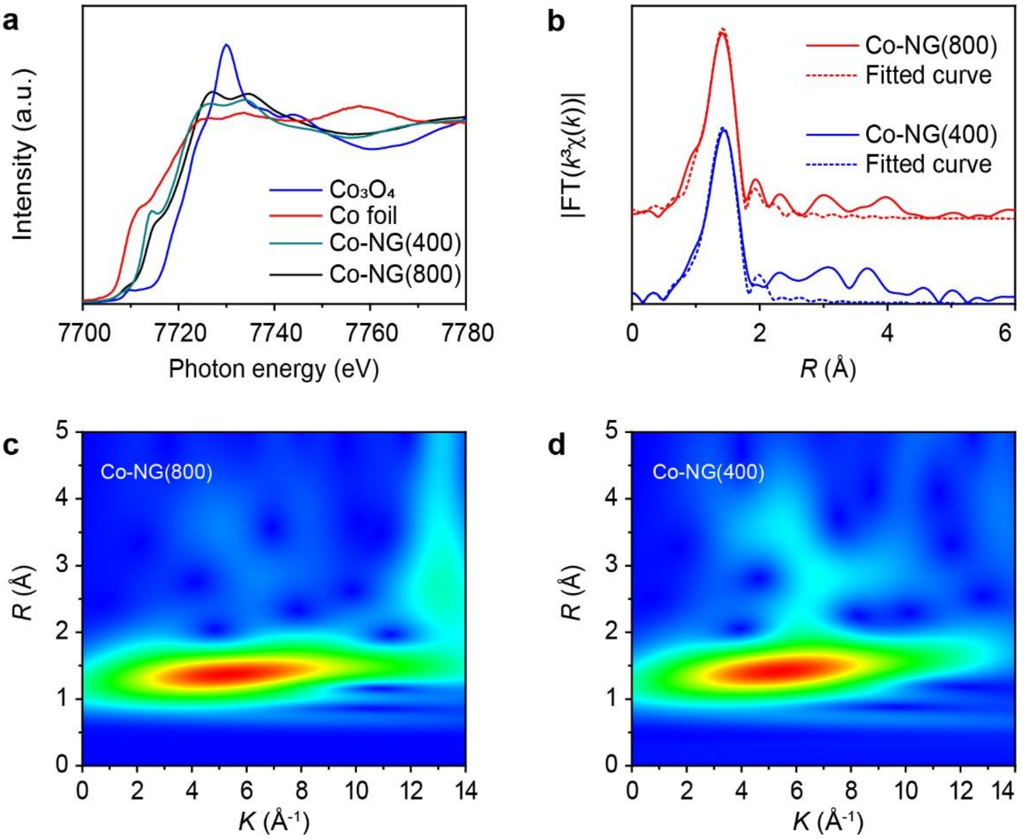

Figure S9. Characterization of the Co-NG(400) electrocatalyst. a) Co *K*-edge XANES spectra recorded for the Co-NG(400), Co-NG(800), Co_3O_4, and Co foil (indicated). b) FT spectra obtained from the k^3-weighted EXAFS data and the corresponding fitted curves for the Co-NG(400) and Co-NG(800) (indicated). c,d) WT contour plots of the EXAFS for: c) Co-NG(800) and d) Co-NG(400).

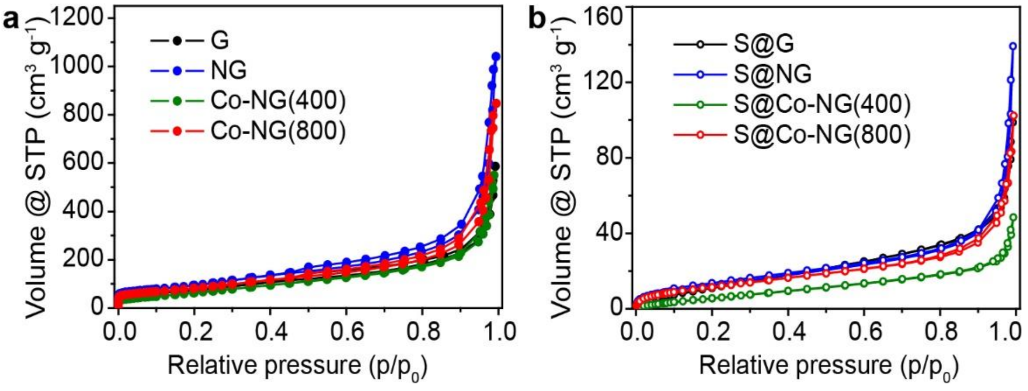

Figure S10. N_2 adsorption/desorption isotherms recorded for G, NG, Co-NG(400), and Co-NG(800). (a) Before the incorporation of sulfur, and (b) after the incorporation of sulfur.

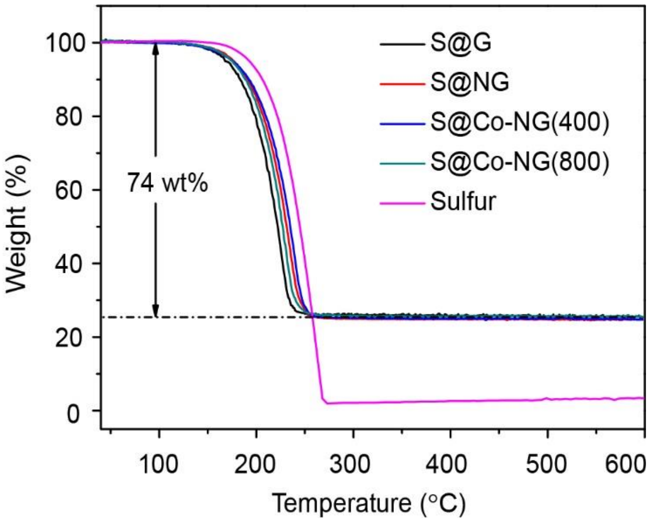

Figure S11. TGA data recorded for the S@Co-NG(800), S@Co-NG(400), S@NG, and S@G composites and sulfur as obtained under an atmosphere of argon (indicated).

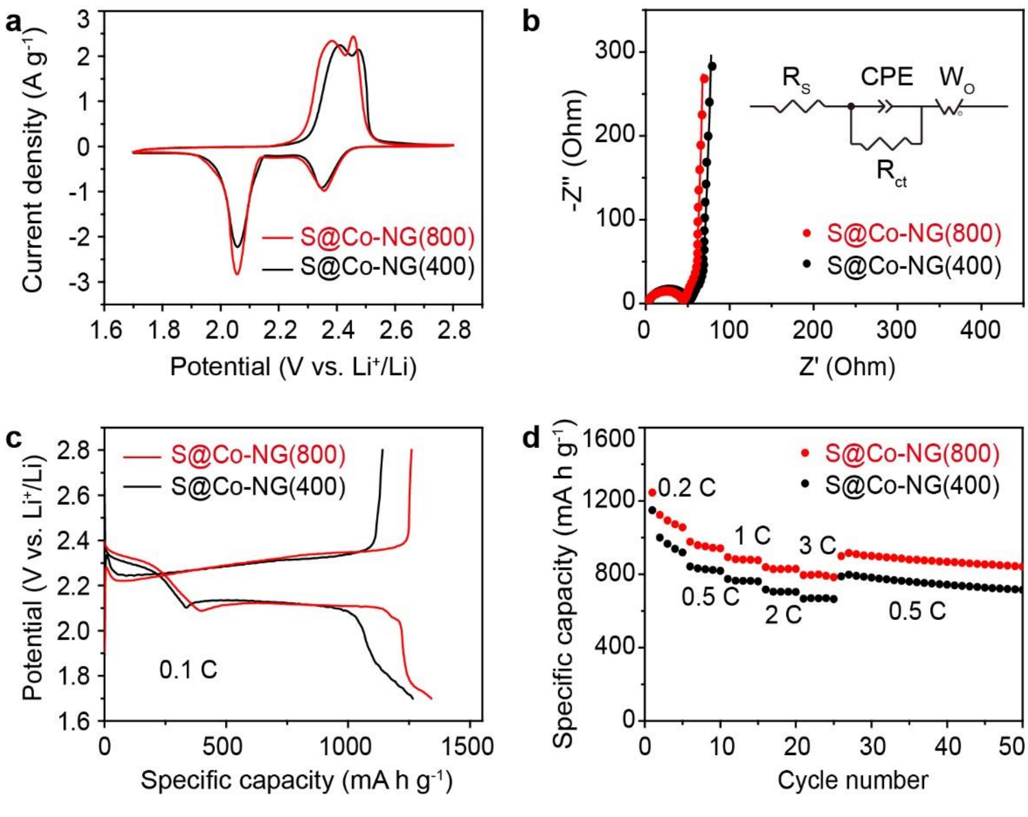

Figure S12. Electrochemical characterization of Li−S cells. a) CV curves recorded at a scan rate of 0.1 mV s^{-1} for the Li−S cells prepared from S@Co-NG(800) and S@Co-NG(400). b) EIS curves recorded for the Li−S cells prepared from S@Co-NG(800) and S@Co-NG(400) (dots) and the corresponding fitting curve (solid lines). The inset shows the equivalent circuit used to fit the EIS curves. c) Charge/discharge profiles recorded at 0.1 C for the Li−S cells prepared from S@Co-NG(800) and S@Co-NG(400). d) Rate capabilities recorded for the Li−S cells prepared from S@Co-NG(800) and S@Co-NG(400). The specific materials analyzed are indicated in the respective legends.

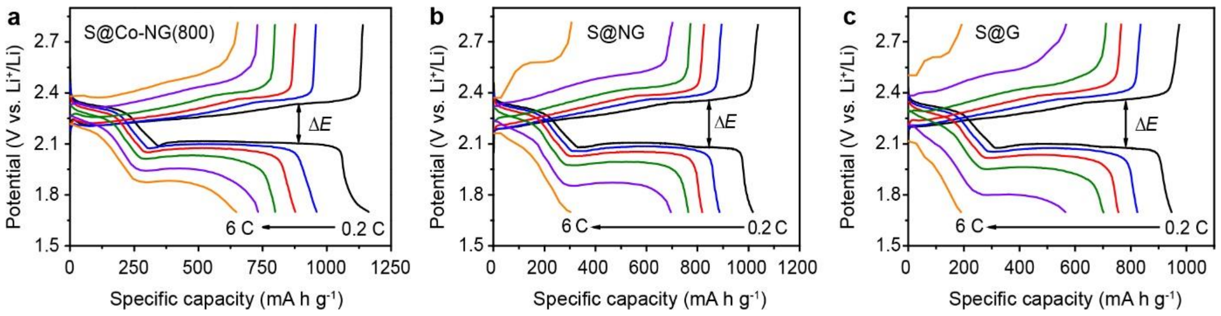

Figure S13. Charge/discharge profiles recorded at 0.2, 0.5, 1, 2, 4, and 6 C for: a) S@Co-NG(800) cathode. b) S@NG cathode. c) S@G cathode.

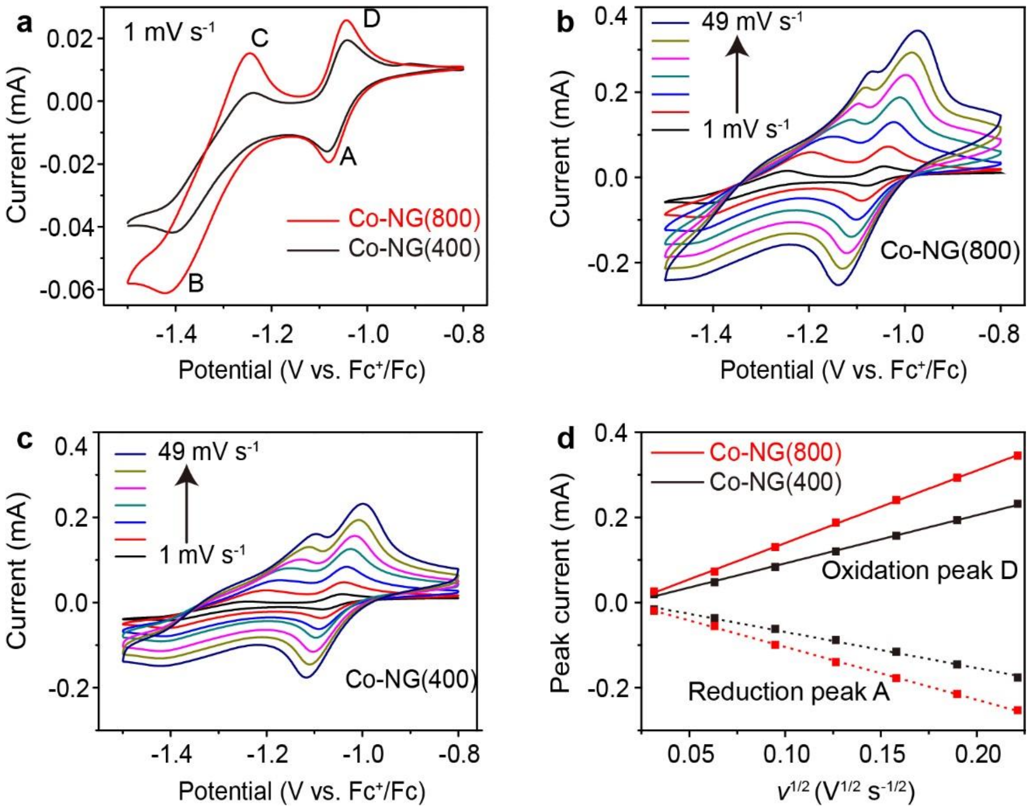

Figure S14. Three-electrode tests for the different electrodes. a) CV curves recorded at a scan rate of 1 mV s^{-1} for Co-NG(800) and Co-NG(400) electrodes. b,c) CV curves recorded at a series of scan rates of 1, 4, 9, 16, 25, 36, and 49 mV s^{-1} for (b) Co-NG(800) electrode, (c) Co-NG(400) electrode. d) Plots of I_p values, derived from the oxidation peak D and the reduction peak A, as function of $v^{1/2}$ for the different electrodes. The specific materials analyzed are indicated in the respective legends.

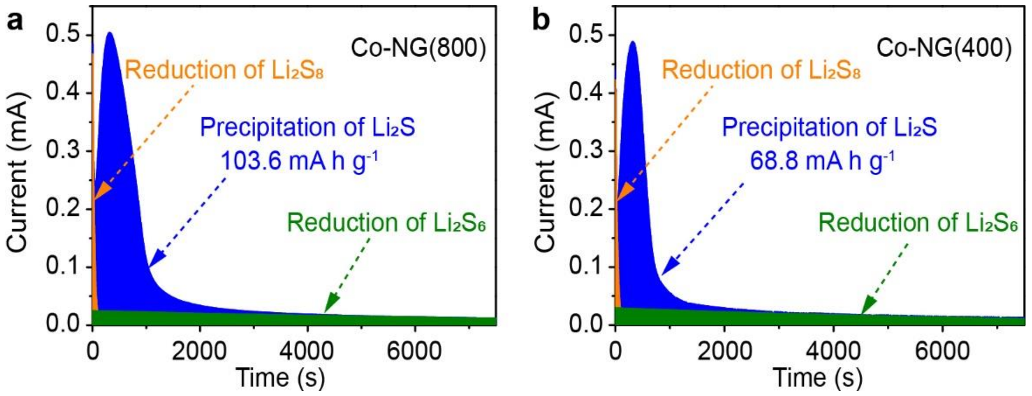

Figure S15. Potentiostatic Li_2S precipitation recorded for: a) Co-NG(800) electrode. b) Co-NG(400) electrode.

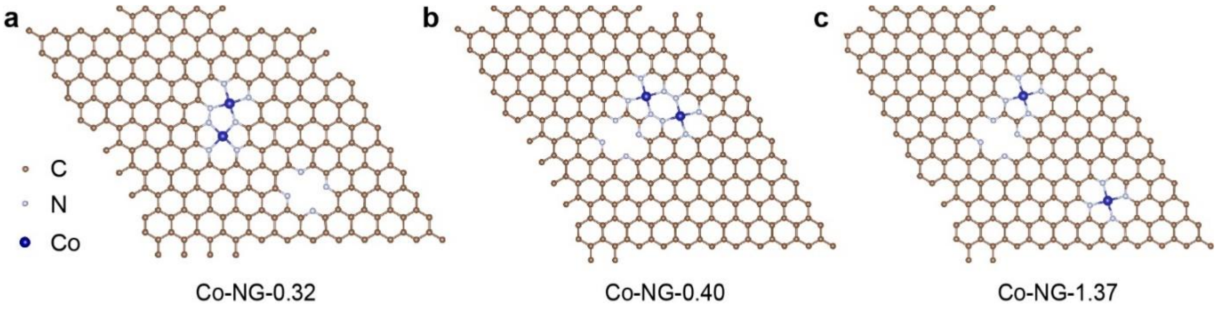

Figure S16. Three representative substrates with different distances between adjacent Co atom: a) 0.32 nm. b) 0.40 nm. c) 1.37 nm.

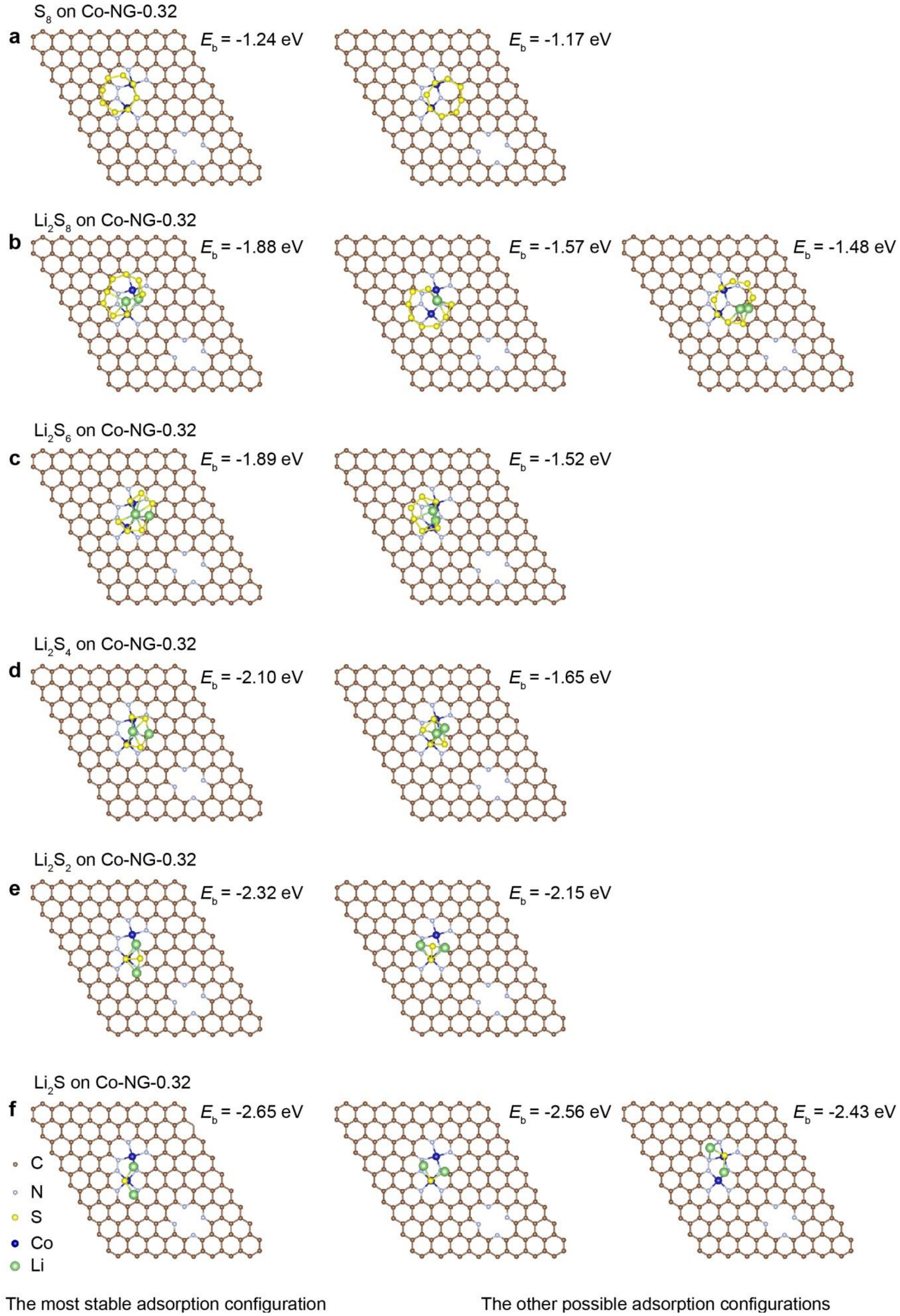

Figure S17. The most stable and other possible adsorption configurations of a variety of sulfur species on Co-NG-0.32. a) S_8 on Co-NG-0.32. b) Li_2S_8 on Co-NG-0.32. c) Li_2S_6 on Co-NG-0.32. d) Li_2S_4 on Co-NG-0.32. e) Li_2S_2 on Co-NG-0.32. f) Li_2S on Co-NG-0.32.

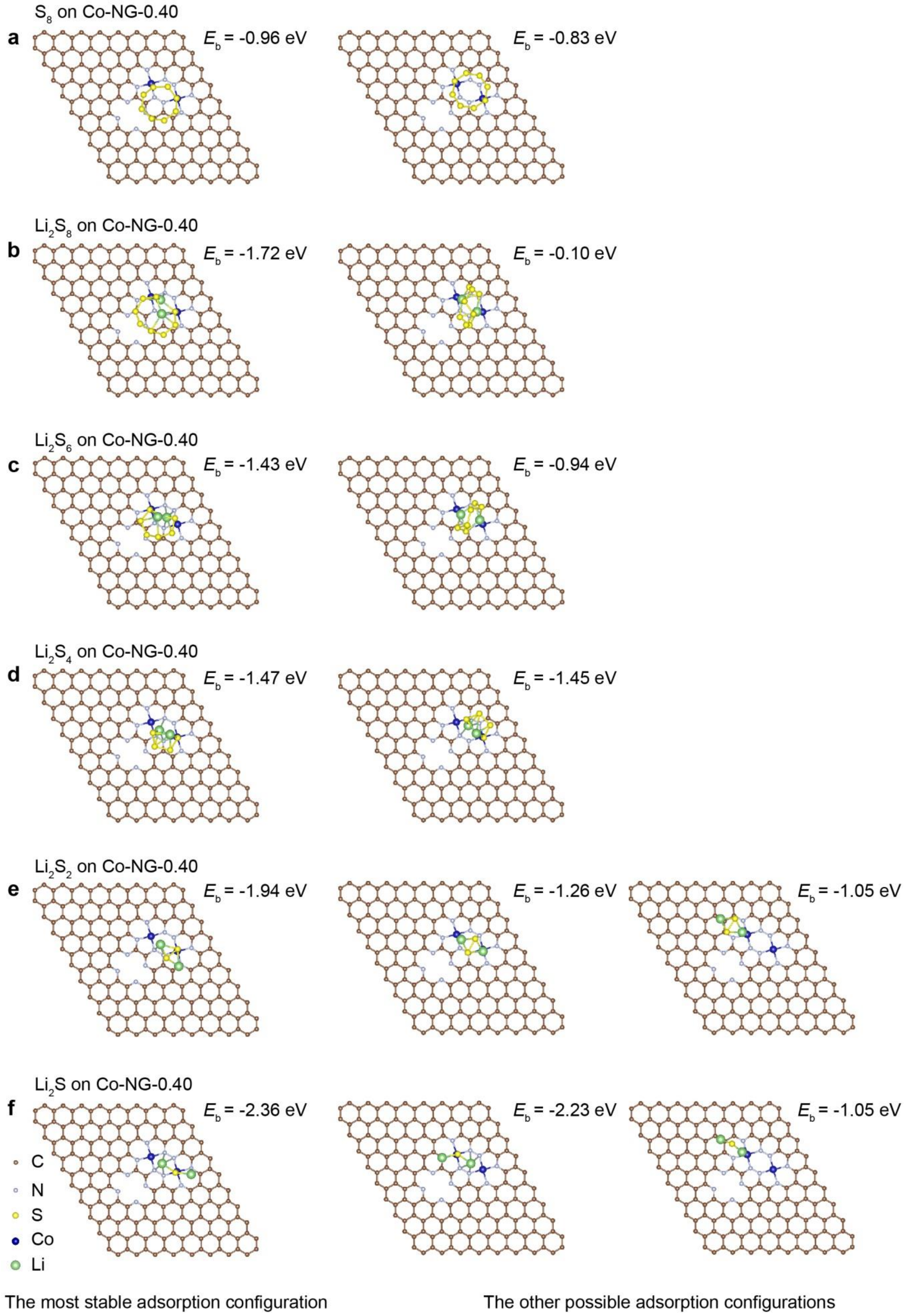

Figure S18. The most stable and other possible adsorption configurations of a variety of sulfur species on Co-NG-0.40. a) S_8 on Co-NG-0.40. b) Li_2S_8 on Co-NG-0.40. c) Li_2S_6 on Co-NG-0.40. d) Li_2S_4 on Co-NG-0.40. e) Li_2S_2 on Co-NG-0.40. f) Li_2S on Co-NG-0.40.

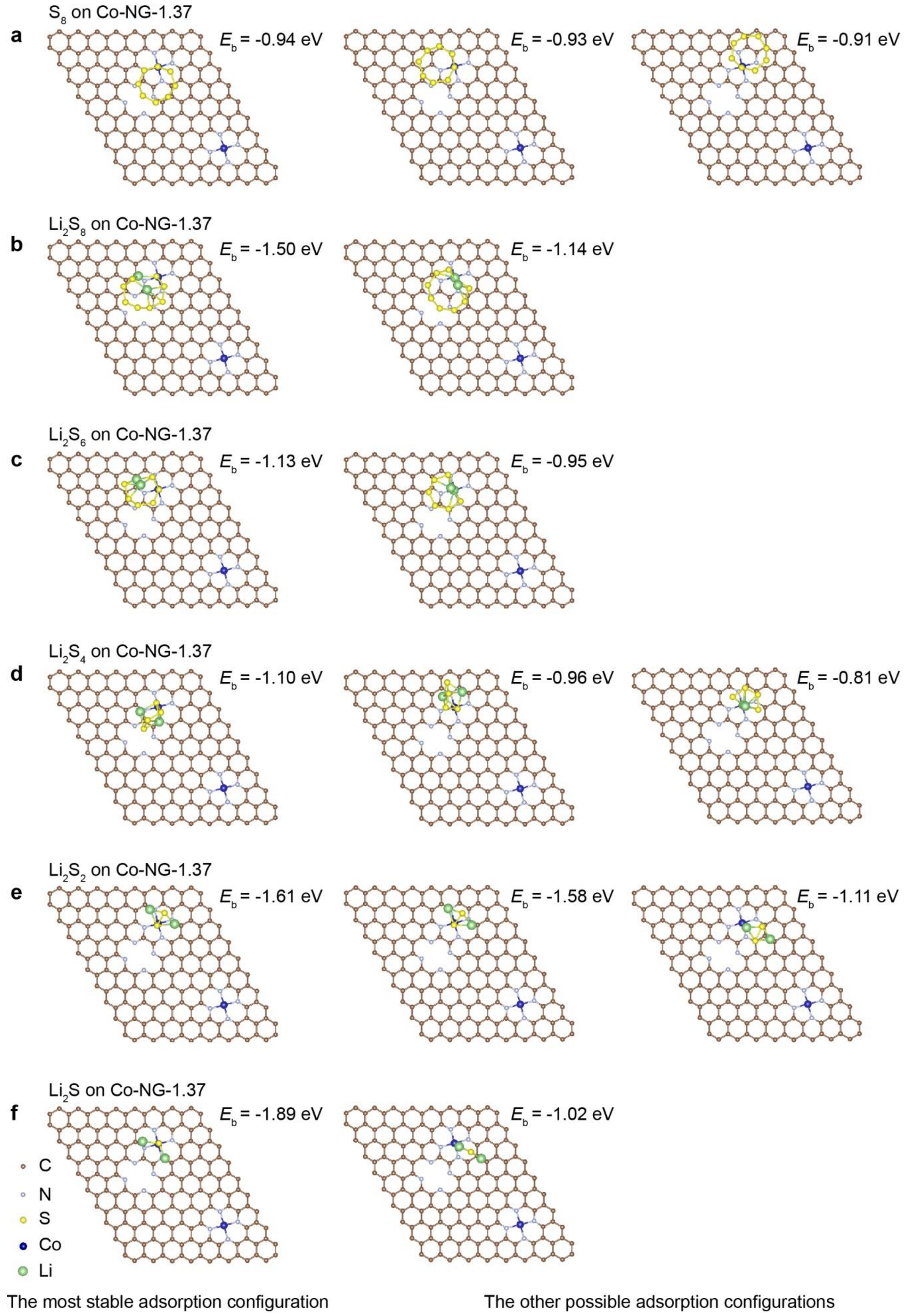

Figure S19. The most stable and other possible adsorption configurations of a variety of sulfur species on Co-NG-1.37. a) S_8 on Co-NG-1.37. b) Li_2S_8 on Co-NG-1.37. c) Li_2S_6 on Co-NG-1.37. d) Li_2S_4 on Co-NG-1.37. e) Li_2S_2 on Co-NG-1.37. f) Li_2S on Co-NG-1.37.

Table S1. Co K-edge EXAFS curve fitting parameters.

Sample	Shell	n	R (Å)	σ^2 (Å^2·10^{-3})	ΔE_0 (eV)	R factor (%)
Co foil	Co–Co	12	2.49	6.1	6.3	0.004
Co-NG(400)	Co–N	3.92	1.91	7.8	1.0	0.433
Co-NG(800)	Co–N	4.11	1.88	7.7	–1.8	0.028

n: coordination number; R: bond distance; σ^2: Debye-Waller factor accounting for thermal and structural disorders; ΔE_0: inner potential correction; R factor (%) indicating the goodness of the fitting. The amplitude reduction factor (S_0^2) was fixed at 0.76.

Table S2. Specific surface areas and pore volume values that were determined for different materials and derived from the curves shown in Figure S10.

Sample	Specific surface area (m^2 g^{-1})	Pore volume (cm^3 g^{-1})
G	280.4	0.92
NG	348.1	1.28
Co-NG(400)	257.4	0.86
Co-NG(800)	305.7	1.17
S@G	48.2	0.15
S@NG	49.7	0.21
S@Co-NG(400)	25.8	0.07
S@Co-NG(800)	46.2	0.16

Table S3. A summary of parameters used to prepare Li–S batteries that contained SAC-based cathodes and corresponding electrochemical performance data.

Material	Sulfur loading (mg cm^{-2})	Current rate (C)	Initial capacity (mA h cm^{-2})	Capacity retention (%)	Ref.
Co-N/G	6.0	0.2	5.1	97	[5]
HFeNG	5.0	0.1	5.0	70#	[8]
Ni@NG	6.0	1	3.54#	85	[9]
SAV@NG	5.0	0.5	3.22	75	[10]
SAFe@g-C_3N_4	2.3	0.2	3.17	90	[11]
Fe_1/NG	4.5	0.5	4.80	84	[12]
**Co-NG(800)**	**11.8**	**0.1**	**10.47**	**85**	**This work**

#) These values are estimated from the figures.

Table S4. A summary of Li-ion diffusion coefficients obtained for different electrodes. These coefficients were derived from the data in Figure 4 and Figure S14 (Supporting Information) using the Randles-Sevcik equation.

Electrodes	Peak A		Peak D	
	Slop	D_{Li}^{+} (cm^2 s^{-1})	Slop	D_{Li}^{+} (cm^2 s^{-1})
G	0.52	4.87×10^{-11}	0.69	8.42×10^{-11}
NG	0.99	1.75×10^{-10}	1.45	3.73×10^{-10}
Co-NG(400)	0.85	1.27×10^{-10}	1.13	2.27×10^{-10}
Co-NG(800)	1.24	2.72×10^{-10}	1.70	5.13×10^{-10}

References

[1] a) M. Vasilopoulou, D. G. Georgiadou, A. M. Douvas, A. Soultati, V. Constantoudis, D. Davazoglou, S. Gardelis, L. C. Palilis, M. Fakis, S. Kennou, T. Lazarides, A. G. Coutsolelos, P. Argitis, *J. Mater. Chem. A* **2014**, 2, 182; b) T. Lazarides, I. V. Sazanovich, A. J. Simaan, M. C. Kafentzi, M. Delor, Y. Mekmouche, B. Faure, M. Réglier, J. A. Weinstein, A. G. Coutsolelos, T. Tron, *J. Am. Chem. Soc.* **2013**, 135, 3095.

[2] J. Geng, H.-T. Jung, *J. Phys. Chem. C* **2010**, 114, 8227.

[3] G. Kresse, J. Furthmüller, *Comput. Mater. Sci.* **1996**, 6, 15.

[4] a) J. P. Perdew, K. Burke, M. Ernzerhof, *Phys. Rev. Lett.* **1996**, 77, 3865; b) G. Kresse, J. Furthmüller, *Phys. Rev. B* **1996**, 54, 11169.

[5] Z. Du, X. Chen, W. Hu, C. Chuang, S. Xie, A. Hu, W. Yan, X. Kong, X. Wu, H. Ji, L.-J. Wan, *J. Am. Chem. Soc.* **2019**, 141, 3977.

[6] M. Dion, H. Rydberg, E. Schröder, D. C. Langreth, B. I. Lundqvist, *Phys. Rev. Lett.* **2004**, 92, 246401.

[7] a) Q. He, B. Yu, Z. Li, Y. Zhao, *Energy Environ. Mater.* **2019**, 2, 264; b) S. Gao, G. Shi, H. Fang, *Nanoscale* **2016**, 8, 1451.

[8] Y. Wang, D. Adekoya, J. Sun, T. Tang, H. Qiu, L. Xu, S. Zhang, Y. Hou, *Adv. Funct. Mater.* **2018**, 29, 1807485.

[9] L. Zhang, D. Liu, Z. Muhammad, F. Wan, W. Xie, Y. Wang, L. Song, Z. Niu, J. Chen, *Adv. Mater.* **2019**, 31, 1903955.

[10] G. Zhou, S. Zhao, T. Wang, S.-Z. Yang, B. Johannessen, H. Chen, C. Liu, Y. Ye, Y. Wu, Y. Peng, C. Liu, S. P. Jiang, Q. Zhang, Y. Cui, *Nano Lett.* **2020**, 20, 1252.

[11] C. Lu, Y. Chen, Y. Yang, X. Chen, *Nano Lett.* **2020**, 20, 5522.

[12] K. Zhang, Z. Chen, R. Ning, S. Xi, W. Tang, Y. Du, C. Liu, Z. Ren, X. Chi, M. Bai, C. Shen, X. Li, X. Wang, X. Zhao, K. Leng, S. J. Pennycook, H. Li, H. Xu, K. P. Loh, K. Xie, *ACS Appl. Mater. Interfaces* **2019**, 11, 25147.
